# Deciphering the heterogeneity of chicken gonadal primordial germ cells via single-cell RNA sequencing

**DOI:** 10.1007/s00018-026-06331-w

**Published:** 2026-07-22

**Authors:** Xiaomin Wang, Shenghu Zhang, Kaipeng Wang, Qianru Li, Yihang Yang, Xingnan Huang, Zhuangfei Wang, Mengmeng Guo, Bo Chen, Zheting Zhang, Bo Wang, Duanqing Pei, Chengchen Zhao

**Affiliations:** 1 Laboratory of Cell Fate Control, School of Life Sciences, Westlake University, Hangzhou, China; 2 Westlake Laboratory of Life Sciences and Biomedicine, Hangzhou, Zhejiang China; 3 Zhejiang University of Science and Technology School of Information and Electronic Engineering, Hangzhou, China; 4 Zhejiang Key Laboratory of Biomedical Intelligent Computing Technology, Hangzhou, China; 5 Fudan University, Shanghai, China

**Keywords:** Avian germline, Gonadal development, Re-established PGC lines, Chimeric chickens, Transcriptomic heterogeneity

## Abstract

Chicken primordial germ cells (PGCs) are valuable for breeding, germplasm preservation, and genetic modifications. Here, using single-cell transcriptome analysis, we found that three transcriptionally distinct PGC subtypes were exist in chicken embryonic gonads PGCs. We further demonstrated that *in vitro*-cultured PGCs could integrate into recipient gonads via blood vessel injection. Additionally, the gene-modified PGCs could be re-established and integrated into recipient gonads after the PGCs were cultured for long-term use by a second blood-vessel injection. Together, these results provide evidence for substantial heterogeneity among chicken gonadal PGCs, which were previously considered a homogeneous cell population. This diversity could be leveraged to establish and expand PGCs for long-term *in vitro* culture from differentiated gonads.

**Supplementary Information:**

The online version contains supplementary material available at https://doi.org/10.1007/s00018-026-06331-w.

## Introduction

Primordial germ cells (PGCs), which form during early development, migrate to the gonads, where they differentiate into functional gametes to transmit genetic information to the next generation. Compared with mammalian PGCs, avian PGCs exhibit distinctive characteristics in terms of specification, migration, and development [1]. In mice, PGCs are specified around embryonic day (E)7 and reach the genital ridges by approximately E10.5 [2]. In chickens (Gallus gallus), PGCs are first detected in the central area pellucida of Eyal-Giladi and Kochav (EGK) stage X embryos [3]. These germ cells are subsequently incorporated into the germinal crescent in Hamburger and Hamilton (HH) stage 4 embryos [4]. They enter the vasculature and circulate at HH10-11 [5], begin to exit the bloodstream by HH15, and migrate along the dorsal mesentery to close to the genital ridges, in which the majority of PGCs settle at HH stage 17 [6, 7]. The population sizes of PGCs also increase gradually from HH stage 10 to HH stage 17, from the blood to the genital ridges [7]. These unique features of chicken PGCs should be investigated in detail to elucidate how PGCs develop in other species that are not easily accessible, such as humans.

The establishment of *in vitro*-cultured chicken PGCs represents a major step toward a molecular understanding of their development. Two major PGCs have been reported to be derived from blood (bPGCs) or gonads (gPGCs) at different stages of development [8–12]. Most of the studies isolated chicken PGCs from blood at early embryonic stages, during which time the PGCs maintained a proliferative and undifferentiated states through different purification techniques [13, 14]. However, starting with only several hundred PGCs collected from blood [7], the propagation of PGCs takes at least several weeks to achieve a final population size of 1 × 10^5^ [15]. Other studies focused on isolating chicken PGCs from the gonads of embryos that were incubated for 5 to 6 days at HH stage 27 to HH stage 30 [11, 16]. After gonadal colonization and sex differentiation, female PGCs undergo a mitotic to meiotic transition, whereas male PGCs enter mitotic arrest. Whether PGCs at these more advanced gonadal stages can be efficiently retrieved and established as long-term cell lines has remained unclear, and relevant technological exploration across avian species has laid an important foundation for germline stem cell derivation [17].

The sex differentiation ability of chicken PGCs after HH stage 30 has yet to be clearly characterized. Single-cell RNA sequencing (scRNA-seq) technology has been used to evaluate chicken embryonic gonadal cells at several developmental stages for the analysis of gonadal sex determination [18, 19] or to identify species-specific characteristics of chicken gonadal PGCs compared with those of zebra finch (*Taeniopygia guttata*) gonadal PGCs [20]. Using scRNA-seq technology, a previous study revealed that zebra finch gonadal PGCs at HH stage 28 can be separated into three subtypes that differ in terms of in apoptosis, proliferation, and high pluripotency. These results provide evidence of heterogeneity in gonadal PGCs, which are considered a single population in birds [21]. Although the selection processes acting on early PGCs are linked to germ cell quality and fertility, insights into the heterogeneity of chicken PGCs remain rather limited.

Here, we performed scRNA-seq on chicken embryonic gonads at stages E7, E8, E9, E10, and E11 (HH stage 31–37) to describe the transcriptome landscapes of PGCs during these stages. We sequenced 103372 gonadal cells and identified 26 clusters, including 11,137 PGCs that segregated into three distinct subpopulations. PGCs were retrieved from stages E7 to E11 for cultivation, and cell lines were established from E7 gonadal PGCs, followed by tdTomato labeling. These PGCs can be cultured long term *in vitro* and integrated into the recipient gonads by blood vessel injection. Afterward, we performed a second blood-vessel injection, in which the PGCs derived from recipient gonads at stage E7 were purified and proliferated *in vitro* and then injected into the blood vessels of another recipient. Our results establish an innovative conceptual framework for understanding the heterogeneous states of chicken PGCs, with implications for optimizing ex *vivo* culture systems and genome editing strategies.

## Results

### Molecular profiling of chicken PGCs at single-cell resolution

**Fig. 1:**
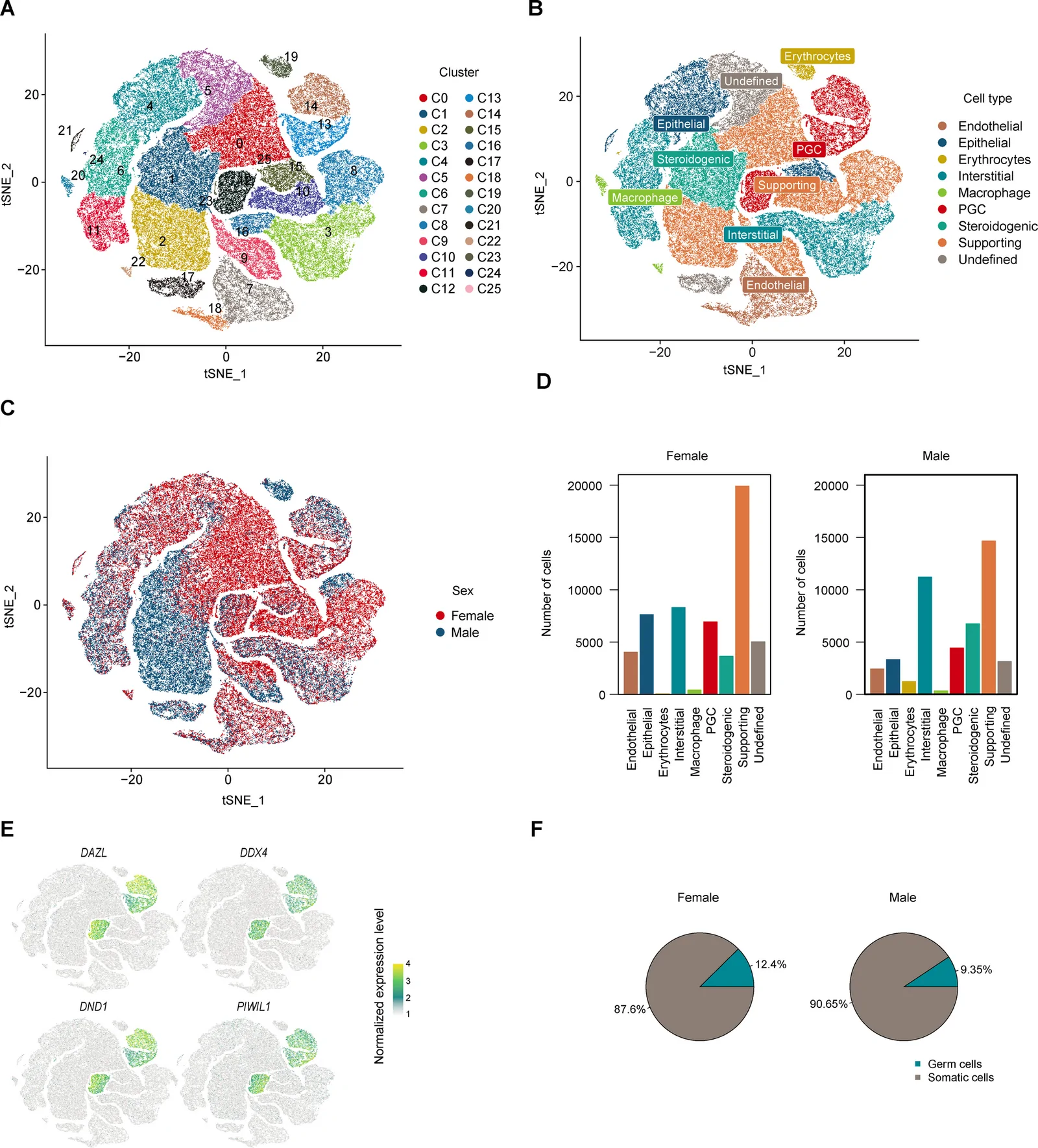
Single-cell transcriptomic atlas of chicken gonads from stages E7 to E11. **A** t-SNE plot showing the 103,372 gonadal cells collected from stage E7 to stage E11 chicken embryos. Each dot represents one cell, and each transcriptional cluster is shown in a different color and labeled with a cluster number. In total, 26 clusters were identified. **B** t-SNE plot showing the cell type annotation of the clusters. The major gonadal cell types, including endothelial, epithelial, erythrocyte, interstitial, macrophage, PGC, steroidogenic, supporting, and undefined cells, were annotated on the basis of canonical marker gene expressions. **C** t-SNE plot showing the distribution of female and male gonadal cells. Each dot represents one cell and is colored according to sex. **D** Bar plots showing the numbers of female and male gonadal cells assigned to each major gonadal cell type. **E** Feature plots showing the normalized expressions of canonical PGC marker genes, including *DAZL*, *DDX4*, *DND1*, and *PIWIL1*, across all the gonadal cells. PGC marker-positive cells were distributed mainly in clusters C12, C13, and C14. **F** Pie charts showing the proportions of germ cells and somatic cells among female and male gonadal cells

Primordial germ cells (PGCs) hold the key to understanding the reproductive process and the pluripotent state of a species. Although PGCs have been characterized in many species, the molecular characteristics of chicken PGCs remain poorly understood. To ensure the inclusion of samples from both sexes, whole gonadal cells were harvested at stage E7 (two replicates, *n* = 8 per replicate); stage E8 (*n* = 8); stage E9 (two replicates, *n* = 8 per replicate); stage E10 (*n* = 8); and stage E11 (*n* = 8) (Supplementary Fig. S1A), and single-cell suspensions were prepared using the Singleron Matrix® Single-Cell Processing System. Across all stages, we profiled 103,372 gonadal cells and identified 11,137 PGCs (Supplementary Fig. S1B). Unsupervised clustering resolved 26 clusters (C0–C25) visualized by the t-distributed stochastic neighbor embedding (t-SNE) algorithm (Fig. 1A). In accordance with the previously established cell type classification criteria for chicken species [19, 22], we assigned nine major cell types, namely, PGCs, epithelial cells, interstitial cells, endothelial cells, supporting cells, erythrocytes, macrophages, and steroidogenic cells (Fig. 1B; Supplementary Fig. S1C).

We next examined how the developmental stage contributed to these clusters. Stage-resolved cluster composition analysis revealed that cells from stages E7 to E11 were unevenly distributed across the 26 clusters, with several clusters showing stage-biased compositions (Supplementary Fig. S1D). We then assessed the distributions of cells assigned to female or male origin. Cells from both sexes were broadly represented across the gonadal cell atlas (Fig. 1C; Supplementary Fig. S1E). Supporting cells, steroidogenic cells, interstitial cells, epithelial cells, and PGCs comprised the major cell populations among both female- and male-origin cells (Fig. 1D). To further examine the sex-associated molecular differences, we performed differential expression analysis between female- and male-origin cells within the major gonadal cell types, including PGCs and somatic populations (Supplementary Fig. S1F).

Notably, the PGCs were heterogeneous and grouped into three transcriptional clusters (e.g., C12, C13, and C14). Canonical PGC markers (e.g., *DAZL*, *DDX4*, *DND1*, and *PIWIL1*) were broadly expressed across these clusters (Fig. 1E). Cells of both female and male origin were present in all three PGC clusters (Fig. 1C and D). The proportions of PGCs were comparable between male- and female-origin cells, representing 9.35% and 12.40% of all sequenced gonadal cells, respectively (Fig. 1F).

### Transcriptional heterogeneity of chicken gonadal PGCs

**Fig. 2:**
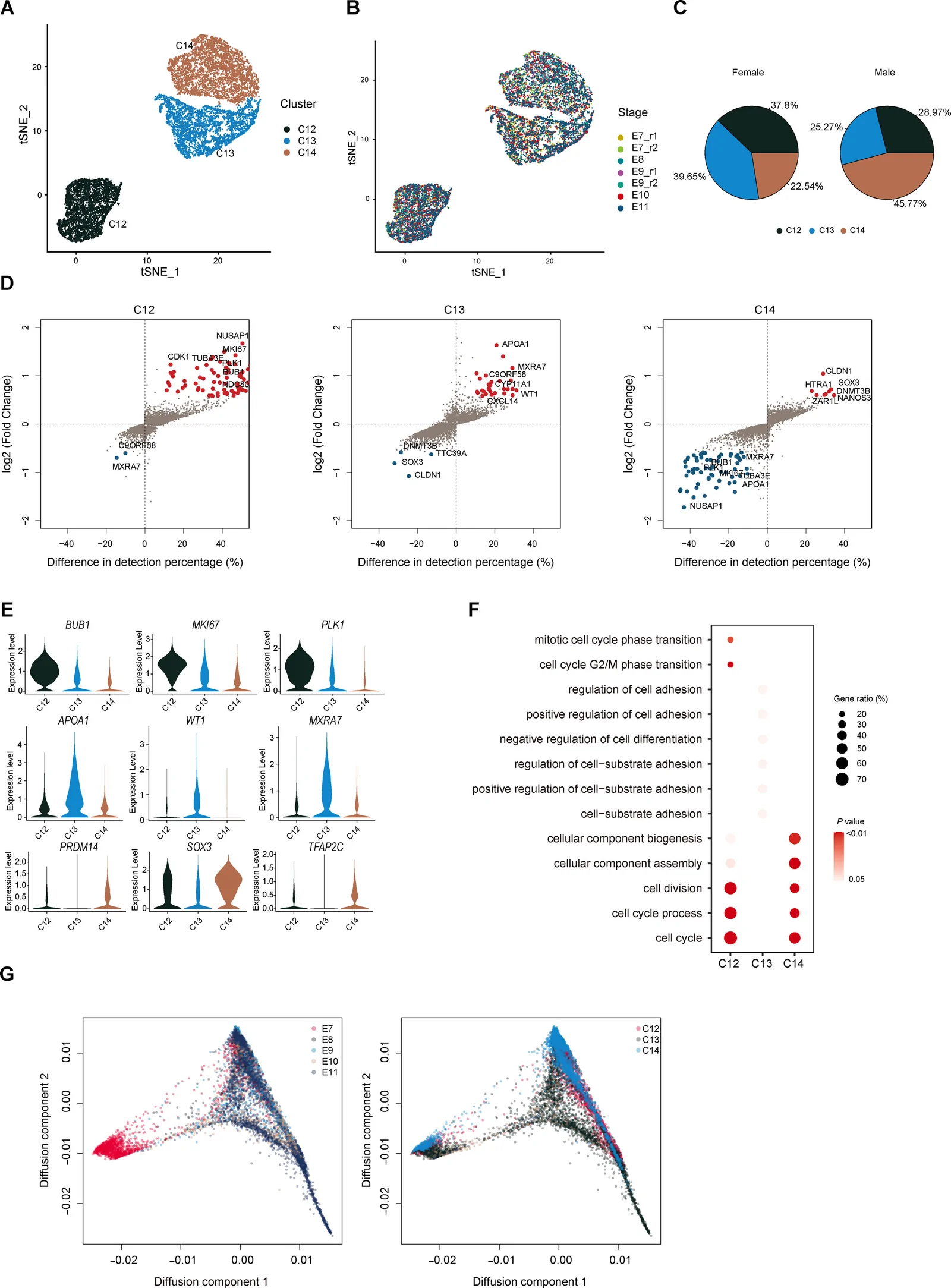
Three transcriptionally distinct PGC subpopulations in developing chicken gonads. **A** t-SNE plot showing PGCs highlighted from the original whole-gonad t-SNE embedding. Each dot represents one cell, and the cells are colored according to the three PGC subclusters C12, C13, and C14. **B** t-SNE plot showing the distributions of PGCs from different developmental stages across the three PGC subclusters. Each dot represents one PGC and is colored according to sample stage. **C** Pie charts showing the sex-stratified compositions of PGC subclusters in female and male gonadal cells. The percentages indicate the relative proportions of C12, C13, and C14 within each sex. **D** Dot plots showing differentially expressed genes for each PGC subcluster. Red dots indicate upregulated genes in this subcluster (average log fold change > 0.58 and differential expression percentage > 10%), blue dots indicate downregulated genes (average log fold change < −0.58 and differential expression percentage < −10%), and gray dots indicate genes without marked differential expressions. Selected representative genes are labeled. **E** Violin plots showing the normalized expressions of representative genes that are associated with C12, C13, and C14. Each violin shows the normalized gene-expression distribution across cells in each PGC subcluster. **F** Dot plot showing representative Gene Ontology terms enriched among subcluster-specific genes. The dot sizes indicate the gene ratios, and the dot colors indicate the adjusted P values. **G** Diffusion map embedding of PGCs. Cells are colored by developmental stage in the left panel and by PGC subcluster in the right panel, showing stage-associated ordering of the three PGC subclusters

We next examined the three PGC clusters in more detail. Visualization of the PGC populations revealed clear separation of C12, C13, and C14 in the t-SNE embedding (Fig. 2A). All three PGC clusters were observed across developmental stages from E7 to E11 rather than being restricted to a single stage (Fig. 2B; Supplementary Fig. S2A). In total, 11,137 PGCs were captured, including 4,381 male-origin cells and 6,756 female-origin cells. Among the male-origin PGCs, 28.97%, 25.27%, and 45.77% were assigned to C12, C13, and C14, respectively; among the female-origin PGCs, 37.80%, 39.65%, and 22.54% were assigned to C12, C13, and C14, respectively (Fig. 2C).

We further compared the gene expressions between female- and male-origin cells within each PGC cluster. We detected sex-biased expression patterns, including female-enriched *HINTW* and *ATP5A1W* in C12 and C14, male-enriched *SOX3* in C12, and female-enriched *HINTW* in C13 (Supplementary Fig. S2B). We also examined stage-associated changes in the relative abundances of the three PGC clusters. In both male- and female-origin PGCs, the C12 population peaked at stage E8 and then steadily declined through stage E11, whereas the proportion of C13 decreased from stage E7 to E8 and then increased from stage E8 to E11. The C14 proportions differed across stages, increasing from stage E7 to E9 and then decreasing from stage E9 to E11 in male-origin PGCs but increasing from stage E7 to E8 and then decreasing from stage E8 to E11 in female-origin PGCs (Supplementary Fig. S2C, S2D). Together, these results indicate that PGC subtype compositions show stage-associated and sex-associated variations.

To further characterize these PGC subpopulations, we identified 79, 34, and 72 cluster-enriched genes for C12, C13, and C14, respectively (Fig. 2D). Representative enriched genes included *BUB1, MKI67, PLK1, CDK1,* and *NUSAP1* in C12; *APOA1*, *WT1*, *MXRA7* and *C9ORF58* in C13; and *PRDM14*, *SOX3*, *TFAP2C*, and *CLDN1* in C14 (Fig. 2D, E; Supplementary Fig. S2E). Consistent with these differential expression patterns, proliferation-associated genes, including *BUB1*, *MKI67*, and *PLK1*, were expressed at higher levels in C12 than in the other PGC subtypes, supporting a strong cell cycle-associated transcriptional signature in C12 (Fig. 2E). In C13, genes associated with PGC migration, homing, and cell adhesion, including *WT1*, *APOA1*, and *MXRA7*, were prominently expressed, suggesting that C13 is enriched for cell adhesion- and migration-associated transcriptional features. In C14, pluripotency-associated genes, including *PRDM14*, *SOX3*, and *TFAP2C*, were enriched, indicating a pluripotency-associated transcriptional feature (Fig. 2E). In contrast, the expressions of *BASP1*, *H3F3B* and *NR0B1*, which are related to the negative regulation of metabolic processes and apoptosis, did not clearly differ from those reported in zebra finch [21] (Supplementary Fig. S2F).

We then performed a Gene Ontology (GO) analysis using PGC subtype-specific genes to further examine the transcriptional features of the three subpopulations (Fig. 2F; Supplementary Fig. 2G). GO terms related to “cell cycle”, “cell cycle process”, and “cell division” were enriched in C12- and C14-specific genes. For example, genes associated with the “cell cycle”, including *NUSAP1*, *CDK1*, *SMC2*, and *TOP2A*; genes associated with “cell cycle process”, including *INCENP*, *NDC80*, *NUF2*, and *CENPH*; and genes associated with “cell division”, including *CENPW*, *NUF2*, and *MASTL*, were enriched in these subtypes. In particular, GO terms related to “cell cycle G2/M phase transition” and “mitotic cell cycle phase transition” were more specifically enriched in C12-associated genes. Consistently, proliferation-associated genes, including *BUB1*, *MKI67* and *PLK1*, were expressed at higher levels in C12, supporting a cell cycle-associated transcriptional signature in this subtype (Fig. 2E and F). In contrast, pluripotency-associated genes, including *PRDM14*, *SOX3*, and *TFAP2C*, were enriched in C14, suggesting a pluripotency-associated transcriptional signature of these cells. In C13, GO terms related to “cell-substrate adhesion”, “positive regulation of cell adhesion”, and “regulation of cell adhesion” were enriched, which is consistent with the increased expressions of *APOA1*, *WT1*, and *MXRA7* in this subtype (Fig. 2E and F; Supplementary Fig. S2F). Finally, we used diffusion maps to characterize the stage-associated ordering among the three PGC subtypes. This analysis revealed C12, C13, and C14 near the initial region, with C13 extending toward the terminal region, providing a representation of the stage-associated relationships among the three PGC subtypes (Fig. 2G).

### Establishing long-term stable PGC lines of PGCs cultured *in vitro*

**Fig. 3:**
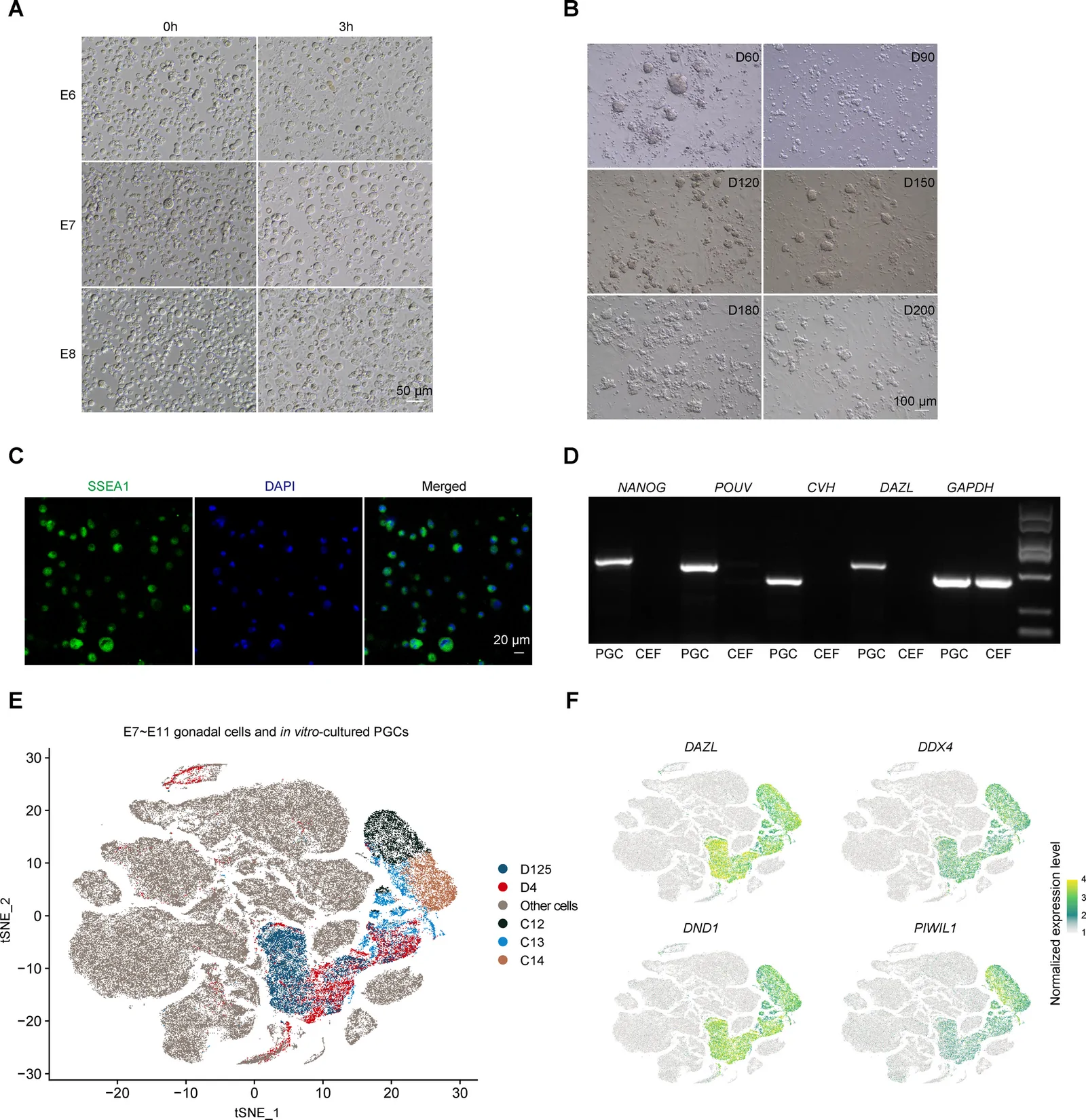
Establishment and molecular characterization of long-term cultured PGC lines from differentiated gonads. **A** Bright-field images of cells derived from stages E6, E7, and E8 gonads at 0 h and 3 h during the initial PGC enrichment step. Scale bar, 50 μm. **B** Bright-field images of long-term *in vitro*-cultured PGCs at the indicated time points. Scale bar, 100 μm. **C** Immunofluorescence staining of *in vitro*-cultured PGCs showing the expressions of PGCs by SSEA1, DAPI, and SSEA1 staining and DAPI staining. Scale bar, 20 μm. **D** RT‒PCR detection of germ cell markers (e.g., *NANOG*, *POUV*, *CVH*, and *DAZL*) in *in vitro*-cultured PGCs compared with those in chicken embryonic fibroblasts. **E** Integrated t-SNE plot showing the *in vivo* gonadal atlas at stages E7 ~ E11 stages (including PGC subclusters and other clusters) together with *in vitro*-cultured PGCs at D4 and D125. Each dot represents one cell. The cultured populations at D4 and D125 map closely to the C13 subcluster. **F** Feature plots showing the normalized expressions of canonical PGC markers (e.g., *DAZL*, *DDX4*, *DND1*, and *PIWIL1*) across integrated *in vivo* and *in vitro* cells

Given the heterogeneity observed *in vivo*, we next tested whether gonadal PGCs from different stages could be maintained long term *in vitro*. We isolated chicken gonads at stages E6, E7, and E8 and dissociated them with 0.05% trypsin–EDTA. After 3 h of differential adhesion, almost all the cells were attached to the bottom of the dish, while the PGC suspension in the medium was transferred to another well, which was coated with a mouse feeder (Fig. 3A). The mean numbers of recovered PGCs did not differ among stages E6, E7, and E8 (Supplementary Fig. S3A); therefore, we established PGC lines primarily from E7 gonads. Multiple PGC lines were generated and maintained for more than 200 days *in vitro* (Fig. 3B; Supplementary Fig. S3B). Immunofluorescence confirmed that the cultured cells expressed the PGC marker SSEA1 (Fig. 3C). To further evaluate the expressions of other markers, chicken PGCs and embryonic fibroblasts were collected for RNA extraction, and the results revealed that only the PGCs expressed the markers “*NANOG*, *POUV*, *CVH*, and *DAZL*” (Fig. 3D). Additionally, the PGCs that were cultured *in vitro* for 4 days (Supplementary Fig. S3C) and 125 days were retrieved for single-cell sequencing. We performed a clustering analysis based on the gene expression levels in 5203 and 8833 cells (Supplementary Fig. S1B), resulting in 9 clusters using the t-distributed stochastic neighbor embedding (t-SNE) algorithm. PGC markers, such as “*DAZL*, *DDX4*, *DND1*, and *PIWIL1*”, were highly expressed in most clusters (Supplementary Fig. S3D, S3E). To verify the cell identities of the PGCs cultured *in vitro*, we compared them *in vivo* and found that the PGCs cultured *in vitro* had the greatest transcriptional similarity to the C13 PGC subpopulation from the gonads (Fig. 3E). Additionally, PGC markers (e.g., *DAZL*, *DDX4*, *DND1*, and *PIWIL1*) were highly expressed in these groups (Fig. 3F). As demonstrated by the above analyses, *in vivo*-derived PGCs, which are composed of three heterogeneous subpopulations, possess the capacity for long-term *in vitro* expansion. Notably, after only four days of *in vitro* culture, the cultured PGC population predominantly showed a transcriptional state similar to that of the C13 subpopulation.

### Integration of PGCs into Gonads

**Fig. 4:**
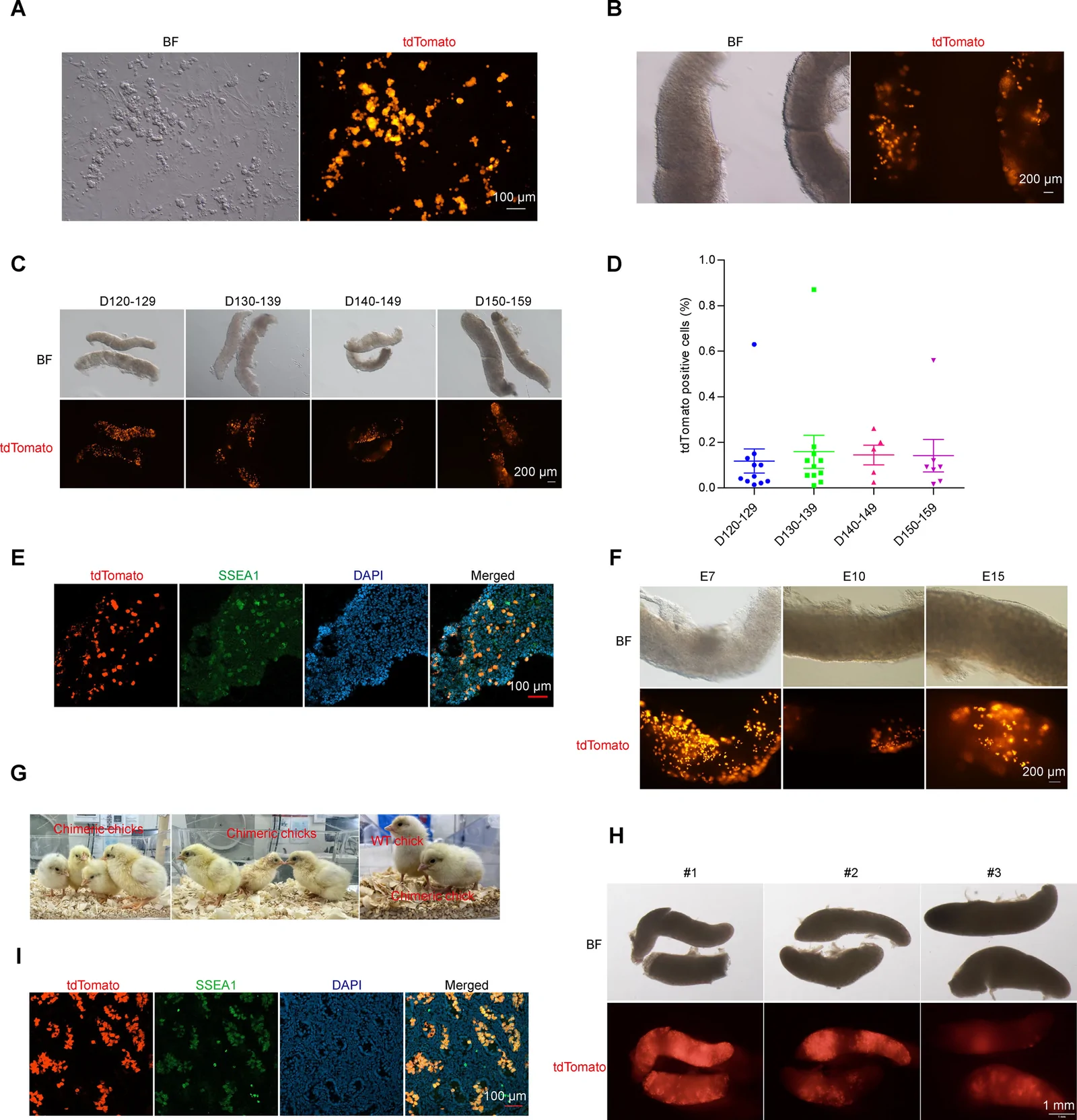
Gonadal localization of long-term cultured tdTomato-labeled PGCs after blood vessel injection. **A** Bright-field and fluorescence images of tdTomato-labeled PGCs cultured *in vitro*. Scale bar, 100 μm. **B** Representative recipient gonads at stage E7 showing tdTomato-positive cells following blood-vessel injection. Scale bar, 200 μm. **C** Representative recipient gonads at stage E7 after injection of PGCs cultured *in vitro* for the indicated durations from 120 to 159 days. Scale bar, 200 μm. **D** Flow cytometry quantification of the contribution of tdTomato-positive cells to recipient gonads at stage E7 after injection of PGCs cultured *in vitro* for the indicated culture-duration (120 days to 159 days). **E** Immunofluorescence of recipient gonads at stage E7 showing tdTomato, SSEA1, and DAPI, and SSEA1 staining for PGC markers and DAPI staining for nuclei. Scale bar, 100 μm. **F** Representative recipient gonads at stages E7, E10, and E15 showing tdTomato-positive cells after blood vessel injection. Scale bar, 200 μm. **G** Representative chimeric chickens and wild-type chicks derived from PGC blood-vessel-injected embryos. WT chicks refer to chicks hatched from eggs without cell injection, whereas chimeric chickens refer to chickens hatched from eggs with PGC injection after 52 h of incubation. **H** Representative male gonads of chimeric chickens imaged by bright field microscopy and fluorescence. Scale bar, 1 mm. **I** Immunofluorescence of P3 chimeric gonads showing tdTomato, SSEA1, and DAPI, and SSEA1 staining for PGC markers and DAPI staining for nuclei. Scale bar, 100 μm

PGC cell lines are valuable because they can serve as the basis for genetic modifications. We therefore labeled cultured PGCs with tdTomato via electroporation (Fig. 4A). The transgenic PGCs could also be cultured long term *in vitro* (Supplementary Fig. S4A). To test gonadal colonization, tdTomato^+^ PGCs were injected into the dorsal aorta of the recipients at 51–53 h of incubation (Supplementary Fig. S4B), and the embryos were analyzed at E7. Fluorescent tdTomato^+^ cells were detected in the recipient gonads, indicating successful colonization (Fig. 4B). The tdTomato-labeled PGCs were cultured *in vitro* for 120 to 159 days and injected into the recipient blood vessels. These PGCs subjected to prolonged *in vitro* culture were subsequently detected in recipient gonads after blood-vessel injection, indicating that long-term cultured PGCs retained the ability to localize to the gonadal region (Fig. 4C). To determine the ratio of chimerism, recipient chicken gonads at stage E7, which were injected with PGCs cultured for 120–129, 130–139, 140–149, or 150–159 days *in vitro* at 51 to 53 h after incubation, were digested into single cells and analyzed by flow cytometry. The chimeric ratios of PGCs cultured *in vitro* for 120–129, 130–139, 140–149, or 150–159 days in whole gonads were 0.12%, 0.16%, 0.14%, and 0.14%, respectively (Supplementary Fig. S4C), with no significant difference between different days cultured *in vitro* from 120 to 159 days (Fig. 4D). Our results demonstrated that the PGCs derived from differentiated gonads could be cultured long term *in vitro* and migrate into the recipient gonads at stage E7 when they were injected into blood vessels at 51 to 53 h after incubation. To determine the cell identity of the integrated PGCs at the stage-E7 gonads, immunofluorescence was carried out to determine the expression of the PGC marker SSEA1. The results demonstrated that most of the SSEA1-positive PGCs were marked with tdTomato (Fig. 4E; Supplementary Fig. S4D). To confirm additional chimerism, the recipient chicken embryos that were injected with PGCs cultured *in vitro* were incubated until stages E10 and E15. The results revealed that the chicken PGCs cultured *in vitro* could also migrate and settle into the gonads (Fig. 4F). To determine the chimerism ratio of the chickens, 45 recipient embryos injected with tdTomato-marked PGCs were incubated (Supplementary Fig. S4E). A total of thirteen chickens were hatched following with three deaths in few hours, including one chimeric and two wild-type chickens. The remaining ten chickens were dissected at 1 to 3 days post-hatching and confirmed to be chimeric (Fig. 4G). For these eleven chimeric chickens, both the male and female chimeric chicken gonads were tdTomato positive (Fig. 4H; Supplementary Fig. S4F). The tdTomato-marked cells were present only in the chimeric chicken gonads, and no tdTomato^+^ cells were detected in other tissues, such as the heart, liver, or lung (Supplementary Fig. S4G). The flow cytometry results revealed that the chimerism ratios of E9 chimeric embryos and chimeric chickens reached 0.27% and 0.69%, respectively (Supplementary Fig. S4H). Immunofluorescence staining of the chimeric chicken gonads also revealed that all the tdTomato^+^ cells were PGCs that were positive for SSEA1 (Fig. 4I; Supplementary Fig. S4I).

### Re-establishing stable PGC lines from injected Gonads

**Fig. 5:**
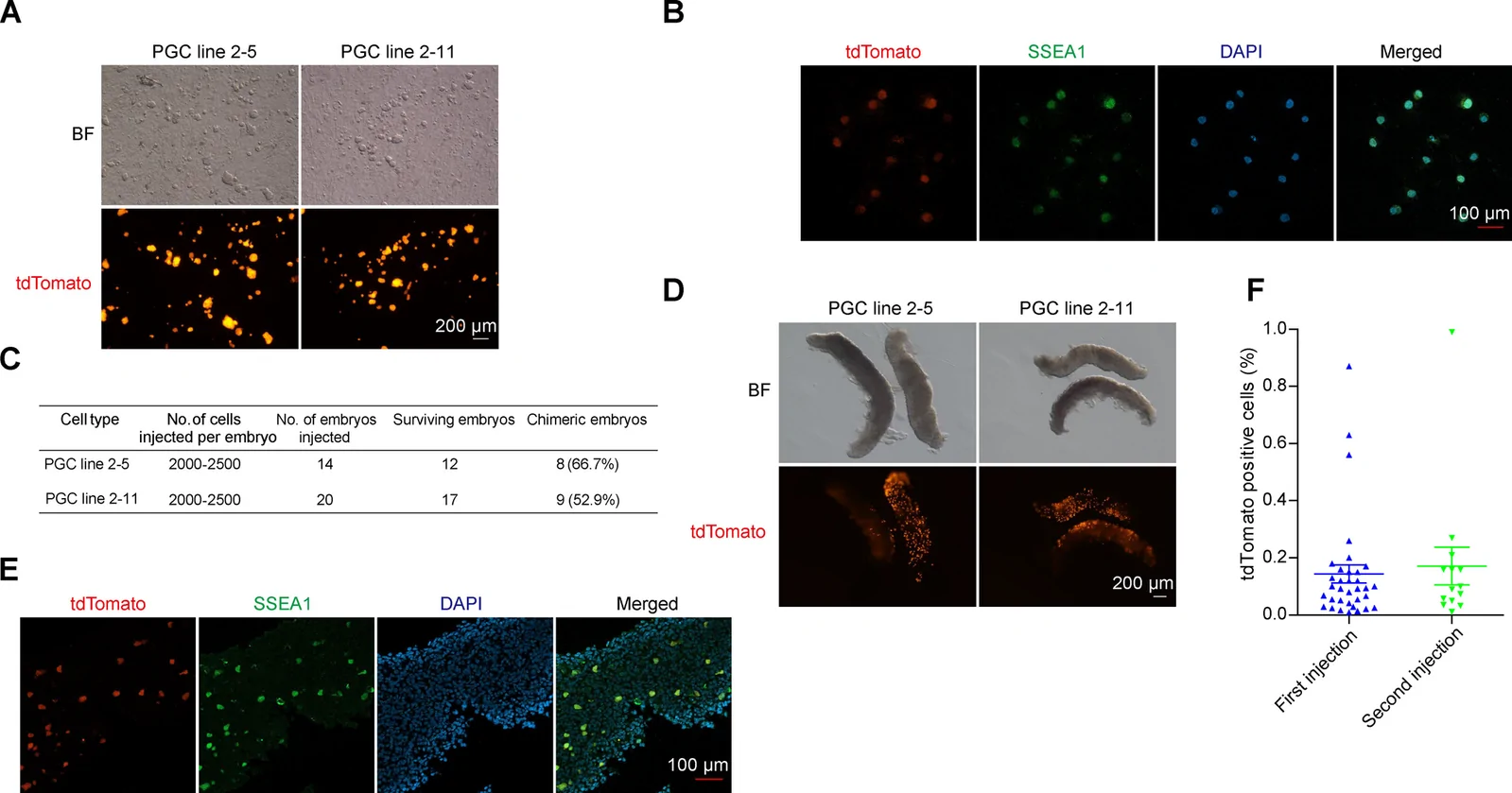
Re-establishment of tdTomato-labeled PGC lines from recipient gonads and secondary blood-vessel injection. **A** Bright-field and fluorescence images of two re-established tdTomato-positive PGC lines derived from two recipient gonads. Scale bar, 200 μm. **B** Immunofluorescence staining of re-established PGCs cultured *in vitro* showing tdTomato, SSEA1 and DAPI, and SSEA1 staining for PGC markers and DAPI staining for nuclei. Scale bar, 100 μm. **C** Summary of two re-establishment PGC lines for secondary transplantation experiments, including injected cell numbers, embryo numbers, survival, and chimerism efficiency. **D** Two lines of re-established chicken PGCs cultured *in vitro* migrated and settled in the embryonic gonads at stage E7; scale bar 200 μm. **E** Immunofluorescence confirmed that the tdTomato-positive cells coexpress SSEA1 in the recipient gonads when the chicken PGCs were reestablished for secondary injection. Scale bar, 100 μm. **F** Flow cytometry quantification of the contribution of tdTomato-positive cells in the stage E7 recipient gonads to the first and second blood vessel injections

To determine whether the donor PGCs remained viable and expandable after the recipient gonads were colonized, we recovered the tdTomato^+^ progeny from the chimeric embryos. Recipients were injected at 51–53 h of incubation and gonads were collected at stage E7. The retrieved PGCs were cultured for 3–4 days, after which the tdTomato^+^ PGCs were enriched by flow cytometry (Supplementary Fig. S5A). Several tdTomato^+^ PGCs lines were established after 2 weeks of expansion (Fig. 5A; Supplementary Fig. S5B). The recovered lines expressed the PGC marker *SSEA1* by immunofluorescence (Fig. 5B; Supplementary Fig. S5C). We then reinjected two reconstructed lines (2,000–2,500 cells per embryo) into new recipients (14 and 20 embryos, respectively). The injected chicken embryos were incubated until stage E7, and the gonads were collected. Twelve and 17 embryos survived, with 8 (66.7%) and 9 (52.9%) showing tdTomato^+^ gonadal colonization, respectively (Fig. 5C). The gonads from the second blood-vessel injection displayed red fluorescence with tdTomato-marked PGCs at stage E7 (Fig. 5D). Immunofluorescence further confirmed that the tdTomato^+^ cells in the recipient gonads coexpressed SSEA1 (Fig. 5E; Supplementary Fig. S5D). To determine the ratio of chimerism, recipient chicken gonads at stage E7, which were injected with secondary PGCs at 51–53 h of incubation, were dissociated into single cells and analyzed by flow cytometry. The mean proportion of tdTomato^+^ cells (PGCs) among the whole gonad cells reached 0.17%, with values reaching 0.99% (Fig. 5F; Supplementary Fig. S5E), indicating that the chimerism rate was not significantly different following secondary blood-vessel injection.

## Discussion

In summary, we profiled the transcriptional landscape of chicken gonadal germ cells from stages E7 to E11 by single-cell RNA sequencing and identified three transcriptionally distinct PGC states. Leveraging these transcriptomic signatures, we established long-term cultured PGC lines from E7-differentiated gonads and evaluated their gonadal colonization capacity after intravascular injection. Using a second-round injection strategy with PGCs recovered from chimeric gonads, the donor contribution reached 0.99% of the tdTomato^+^ cells in the recipient gonads. Recent work has enabled the stable derivation of embryonic stem cells across multiple avian species, providing valuable pluripotent cell resources for the study of avian development and genetic modification [17]. The establishment of long-term expandable chicken PGC lines in the present study complements existing avian ES cell systems, and these two types of cell platforms may together support future mechanistic studies of avian germline development and support diverse strategies for the generation of genetically modified avian models. Overall, our study provides a transcriptomic framework for understanding the heterogeneity of chicken gonadal PGCs and links these *in vivo* PGC states to long-term cultured gonad-derived PGCs.

We found that cells annotated as PGCs on the basis of the expressions of known germ cell markers (e.g., *DAZL*, *DDX4*, *DND1*, and *PIWIL1*) can be classified into three distinct subtypes (Fig. 2A), and this subtype composition is not solely explained by developmental stage from stages E7 to E11 (Fig. 2B; Supplementary Fig. 2A). This differs from the findings of previous studies reporting limited heterogeneity in PGCs derived from early-stage chicken embryos [21]. This discrepancy may be related to the developmental window examined here (embryos incubated for 7–11 days). In the C12 PGC subtype, the genes encoding *BUB1*, *MKI67*, and *PLK1*, which are related to the mitotic cell cycle, were specifically upregulated (Fig. 2E). To further confirm the identity of the C12 PGC subtype, genes related to stage-specific markers were analyzed according to a previous study [19]. Stage-specific markers such as *KLF4A* and *TUBA3E* were highly expressed in the C12 subtype (Supplementary Fig. 2E), supporting C12 as a proliferative PGC subtype. GO analysis revealed that the C13 PGC subtype is enriched for cell adhesion related terms (Fig. 2F), which may contribute to PGC migration and invasion. Notably, the majority of in vitro-cultured PGCs exhibited transcriptional states similar to the C13 PGC subpopulation and could localize to and integrate into embryonic gonads after blood vessel injection (Fig. 4B, F, G). In mice, compared with cells that arrive later, PGCs that arrive first form a larger population, and this asynchronous migration affects proliferation and clonal expansion. C13 PGCs, whose expressions are specifically related to migration and pluripotency, are presumed to be late-arriving in the gonads. We detected pluripotent genes such as *PRDM14*, *SOX3* and *TFAP2C* selectively in C14 (Fig. 2E). Consistent with a previous study on zebra finch PGCs [21], these cells exhibit a pluripotency-associated transcriptional signature. We also observed higher expressions of *CLDN1*, *HTRA1*, *DNMT3B*, *ZAR1*, and *NANOS3* in C14 than in C12 and C13, suggesting that these genes may serve as candidate markers for future enrichment strategies (Supplementary Fig. S2E). Collectively, the features assigned to the PGC subtypes are inferred from the scRNA-seq profiles, marker expressions, and enrichment analyses. These data alone do not establish definitive functional states, and functional validation will be needed in future studies.

Previous studies of blood-derived or early gonad-derived PGCs generally treated chicken PGCs as a homogeneous population and established lines for gene editing and gonadal colonization [9]. In contrast, our scRNA-seq analysis revealed that PGCs from differentiated gonads are heterogeneous and comprise at least three transcriptional states. We successfully derived long-term PGC lines from stage-E7 gonads, performed fluorescent labeling, and demonstrated efficient gonadal colonization after transplantation (Fig. 4A and B), indicating that differentiated gonad-derived PGCs retain capabilities comparable to those of earlier-stage PGCs. Notably, the C13 subtype cells resemble previously described gonad-derived PGC programs and preferentially match the *in vitro*-cultured PGC state. To increase chimerism, we established a secondary transplantation strategy in which PGCs were recovered from recipient gonads following primary injection. Although the chimerism rate was not significantly elevated, compared with the primary injection, the secondary injection resulted in a slight improvement in the chimerism level. These results suggest that PGCs recovered from recipient gonads can be re-expanded *in vitro* and used for subsequent blood-vessel injection. Together with the transcriptional similarity between *in vitro*-cultured PGCs and the endogenous C13 PGC subpopulation, these findings support the feasibility of recovering and reusing donor-derived PGCs for secondary transplantation. These findings provide a resource and conceptual framework for studying avian germ-cell development *in vivo* and *in vitro*.

The results of our scRNA-seq analysis provide a deeper understanding of chicken PGCs by revealing for the first time the heterogeneity and intrinsic changes in gonadal PGCs, which have hitherto been considered a more convenient source of PGCs for *in vitro* culture. We believe that the single-cell-level profiling results will be useful for future investigations of selection on variation and collection in avian germ cells during embryonic development.

## Materials and methods

### Experimental animals and animal care

All procedures involving chickens were approved by the Institutional Animal Care and Use Committee of Westlake University, Hangzhou, China (Animal Protocol No.23–132-PDQ).

### Gonad collection and dissociation

Gonads were dissected from chicken embryos at stages E6, E7, E8, E9, E10, and E11. The tissues were subsequently washed three times with PBS. Each pair of gonads was pooled in 100 µL of 0.05% trypsin (Gibco, 25300054) and incubated at 37 °C for 8 min with gentle pipetting. Digestion was stopped by the addition of 900 µL of Dulbecco’s modified Eagle’s medium (DMEM; HyClone, SH30022-2B) supplemented with 10% fetal bovine serum (FBS; Gibco, 10099141 C). The resulting single-cell suspensions were used for scRNA-seq or for establishing PGC lines.

### Single-cell library preparation and sequencing

Eight embryonic gonad samples from stages E7, E8, E9, E10, and E11 were dissociated in 100 µL of 0.05% trypsin, followed by the addition of 900 µL of DMEM containing 10% FBS. The samples were subsequently centrifuged at 200 × g for 5 min and resuspended in 1 mL of PBS (Gibco, C10010500BT). *In vitro*-cultured PGCs (4 or 125 days) were collected, centrifuged at 200 × g for 5 min, and resuspended in 1 mL of PBS. The cell suspensions were filtered through 40 μm sterile strainers, centrifuged at 350 × g for 5 min, and resuspended in PBS. Viability was assessed by Trypan blue staining (Invitrogen, T10282) under a light microscope. Single-cell suspensions were loaded onto microwell chips using the Singleron Matrix® Single-Cell Processing System. Barcoding beads were subsequently collected from the microwell chip, after which the mRNA that was captured by the barcoding beads was reverse transcribed to obtain cDNA for PCR amplification. The amplified cDNA was then fragmented and ligated with sequencing adapters. The scRNA-seq libraries were constructed according to the protocol of the GEXSCOPE® Single-Cell RNA Library Kit (Singleron) [23]. Individual libraries were diluted to 4 nM, pooled, and sequenced on an Illumina NovaSeq 6000 with 150-bp paired-end reads.

### Single-cell RNA-seq data analysis

The raw scRNA-seq reads were mapped to the galGal6 reference genome using CeleScope (v2.0.7). Gene-by-cell expression matrices generated by CeleScope were used for downstream analysis in Seurat (v4.3.0) [24]. For each sample, gene expression values were log-normalized, and highly variable genes were identified. Multiple samples were then integrated using the IntegrateData function to reduce sample-specific variations before dimensionality reduction and clustering. After normalization and sample integration, dimensionality reduction was performed using t-distributed stochastic neighbor embedding (t-SNE) analysis. The nearest neighbors of each cell were identified, followed by graph-based clustering using the Louvain algorithm. Dimensionality reduction and clustering were performed in Seurat using the RunTSNE, FindNeighbors, and FindClusters functions. Cluster identities were assigned on the basis of the expressions of canonical marker genes for the major gonadal cell types, including PGCs, supporting cells, steroidogenic cells, epithelial cells, endothelial cells, erythrocytes, macrophages, and interstitial cells. PGC clusters were identified on the basis of the expressions of known germ cell markers, including *DAZL*, *DDX4*, *DND1*, and *PIWIL1*. Gene expressions were visualized using normalized expression values. Feature plots and violin plots were used to show expression distributions on t-SNE embeddings and across clusters, respectively.

Differentially expressed genes were identified for each cluster or PGC subcluster using the FindAllMarkers function in Seurat. Genes whose average log fold change values were > 0.58 and whose differences in detection percentage were > 10% were considered upregulated in the corresponding group. Sex-stratified differential expression analyses were performed between female and male cells within the corresponding cell types or PGC subclusters using the same criteria. Diffusion map analysis was performed using the R package to characterize stage-associated ordering among PGC subclusters. This analysis was used to determine the relationships among PGC subtypes without the assumption of a strict linear developmental trajectory.

### Gene ontology analysis

Functional enrichment analysis was performed using clusterProfiler (v4.6.2) [25]. Gene Ontology terms for each functional cluster are summarized as representative terms. Representative terms were selected for visualization, and enrichment significance is shown as the -log10(P value) in the corresponding figures.

### PGC isolation, purification, and culture

Fertilized eggs (Jing Bai line; Hangzhou Huajie Poultry Industry Co., Ltd.) were incubated at 38˚C and 60% relative humidity. At embryonic Day 7 (HH31), gonads were dissected under a stereomicroscope, and PGCs were enriched by the differential adhesion method to reduce red blood cells and chicken embryonic fibroblasts. PGCs were plated onto mitomycin C-treated mouse embryonic fibroblasts (MEFs) and cultured at 37˚C with 5% atmospheric CO_2_. The culture medium consisted of knockout DMEM (KO-DMEM; Gibco, 10829018) supplemented with 20% FBS; 2% chicken serum (Sigma, C5405); 1% sodium pyruvate (Gibco, 11360070); 1% GlutaMAX (Gibco, 35050061); 1% nonessential amino acids (Gibco, 11140035); 1 × nucleosides (Millipore, ES-008-D); 0.1 mM β-mercaptoethanol (Sigma, M3148); 3 ng/mL human stem cell factor (R&D, 455-MC-020); and 2 ng/mL basic fibroblast growth factor (R&D, 233-FB-025). The medium was changed daily. For passaging, the cells were dissociated with 0.05% trypsin every 3–4 days and replated onto freshly prepared feeder cells.

### Generation of tdTomato-marked PGCs

PGCs were transfected with a tdTomato PiggyBac system for subsequent transplantation. Cells were resuspended in 100 μL electroporation buffer containing 2 μg PBase and 4 μg PB-CAG-tdTomato plasmids (Lonza Mouse Embryonic Stem Cell Nucleofector™ Kit; VPH-1001). The mixture was transferred to electroporation cuvettes and electroporated using the program recommended for mouse embryonic stem cells. Cells were immediately transferred to 1 mL of prewarmed PGC media and plated in a 12-well plate on feeder cells. At 12 h posttransfection, the cells were washed and replated onto fresh feeder cells. After 8 days, the tdTomato^+^ PGCs were enriched by flow cytometry and expanded until blood vessel injection.

### Blood vessel injection and detection of tdTomato-marked PGCs in recipients

For PGC transplantation into recipient embryos, a small window was opened at the pointed end of the recipient egg. A 4-μL suspension containing 2,000–2,500 PGCs was injected into the dorsal aorta using a 40-μm needle at 51–53 h of incubation (HH stage 13–15). The egg was sealed with paraffin film and incubated until stage E7, E8, E9, E10, E11, or E15. After being transferred into the recipient embryos, tdTomato-expressing PGCs were detected in the gonads of the recipients after 7, 8, 9, 10, 11, and 15 days of incubation or after they hatched. Recipient gonads were dissected at the indicated stages, and tdTomato^+^ cells were imaged using a fluorescence microscope (Nikon; Ti2-U).

### Re-establishment of tdTomato-marked PGC lines and second blood-vessel injection

Recipient gonads containing tdTomato-marked cells were collected at stage E7, dissociated into single cells, and plated onto mouse feeder cells. After 3–4 days, the tdTomato^+^ PGCs were enriched by flow cytometry. The tdTomato^+^ PGCs were cultured *in vitro* for proliferation. Expanded tdTomato^+^ PGCs were then injected into the recipient embryos (HH stage 13–15) for second-round blood-vessel injection. Recipient embryos were incubated until stage E7 and assessed by fluorescence imaging.

#### RT‒PCR

*In vitro*-cultured PGCs were harvested, and total RNA was extracted using a FastPure Cell/Tissue Total RNA Isolation Kit V2 (Vazyme, RC112-01). cDNA was synthesized using HiScript II Q RT SuperMix for qPCR (Vazyme, R222-01). PCR was performed using primers for chicken *NANOG*, *POUV*, *CVH*, and *DAZL*. *GAPDH* served as the internal control. The cycling conditions were 35 cycles of 95 °C for 30 s, 60 °C for 30 s, and 72 °C for 1 min. PCR products were visualized using nucleic acid dye (Goldview, DH392-5). The primer sequences used are listed in Supplementary Table S1.

### Flow cytometry analysis and sorting

Gonads from E7 recipient embryos were dissociated into single cells using 0.05% trypsin–EDTA and filtered through a 40 μm strainer. The samples were analyzed on a CytoFLEX LX 5L3 cytometer (Beckman) to quantify the tdTomato^+^ cells. For sorting, the tdTomato^+^ PGCs were collected on a Sony MA900 sorter (Sony, MA900-1) and expanded for subsequent experiments. The data were analyzed with FlowJo software.

### Immunofluorescence analysis of PGCs and gonads

Gonads were fixed in 4% paraformaldehyde (Beyotime, P0099–500 mL) at 4℃ for 4 h, cryoprotected in 30% sucrose (Sigma, V900116) overnight at 4℃, embedded in optimal cutting temperature (OCT) compound (Sakura, 4583), and sectioned at 8–10 μm using a Leica cryostat (Leica, CM3050S). For immunofluorescence, *in vitro–*cultured PGCs were collected by gently shaking the culture dish and then digested into single cells with trypsin (0.05%), after which the cells were added to the slides and air-dried. Sections or PGCs on slides were fixed in 4% paraformaldehyde at room temperature for 15 min, permeabilized with 0.1% Triton X-100 (Beyotime, P0096–100 mL) in PBS for 30 min, and blocked in PBS containing 3% bovine serum albumin (Sigma, SRE0098) for 1 h. Samples were incubated with anti-SSEA1 antibody (Santa Cruz, sc-21702) overnight at 4 ℃, followed by Alexa Fluor® 488-conjugated goat anti-mouse secondary antibody (CTS, 4408S) for 1 h at room temperature. The nuclei were counterstained with DAPI (Beyotime, C1005). Images were acquired on a confocal laser scanning microscope (Zeiss, LSM900).

### Statistical analysis

Statistical analyses were performed in GraphPad Prism (GraphPad Software, La Jolla, CA, United States). Two-group comparisons were conducted using two-tailed Student’s t tests.

## Supplementary Information

Below is the link to the electronic supplementary material.

Supplementary file1 (DOCX 23 KB)

Supplementary file2 (PDF 58126 KB)

## Data Availability

The scRNA-seq data have been deposited in the Gene Expression Omnibus database under the accession number GSE276297. Other data will be made available on request.
