# Supplementary material for: Deciphering the heterogeneity of chicken gonadal primordial germ cells via single-cell RNA sequencing
Source: Cell Mol Life Sci. 2026 Jul 22;83(1):387. doi: 10.1007/s00018-026-06331-w (PMC13680580; doi:10.1007/s00018-026-06331-w)

Figure S1

A

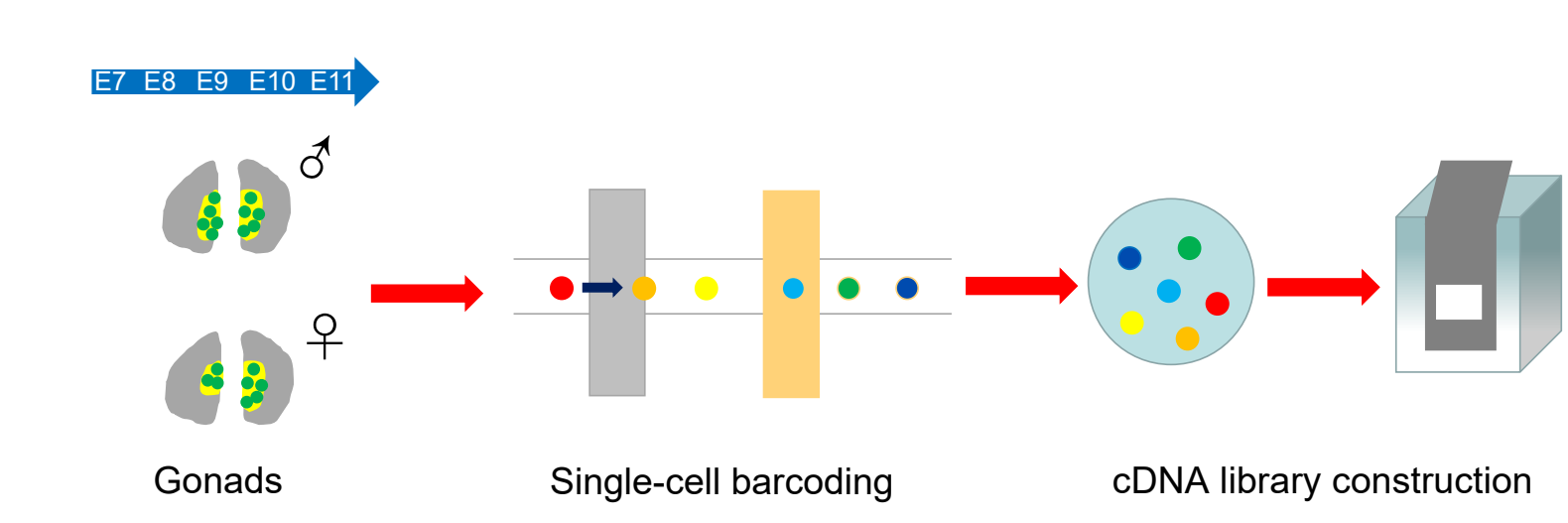

B

Samples	Raw reads	Valid reads	Uniquely mapped reads	% of base pairs in exonic regions	% of base pairs in intronic regions	% of base pairs in intergenic regions	Estimated cells	Total cells	PGCs
E7-r1	601,312,993	317,972,678	277,388,103	86.28	2.11	11.6	18,549	32,385	2,375
E7-r2	706,399,870	549,682,984	498,036,339	85.34	2.93	11.73	13,836		
E8	760,955,944	535,367,560	480,355,466	86.19	3.54	10.27	11,257	11,257	564
E9-r1	701,590,297	633,868,558	541,197,430	78.24	7.36	14.4	12,085	23,629	2,063
E9-r2	762,627,466	538,996,509	490,934,750	84.15	3.99	11.86	11,544		
E10	745,693,077	589,899,274	526,404,623	84.14	4.08	11.78	13,623	13,623	2,086
E11	755,578,268	681,972,885	588,984,160	80.09	6.41	13.5	22,478	22,478	4,049
E7-D4	593,174,084	306,610,973	256,638,941	83.87	3.37	12.76	6,488	6,488	5,203
E7-D125	354,743,565	320,865,555	240,135,781	80.11	2.51	13.42	8,914	8,914	8,833

D

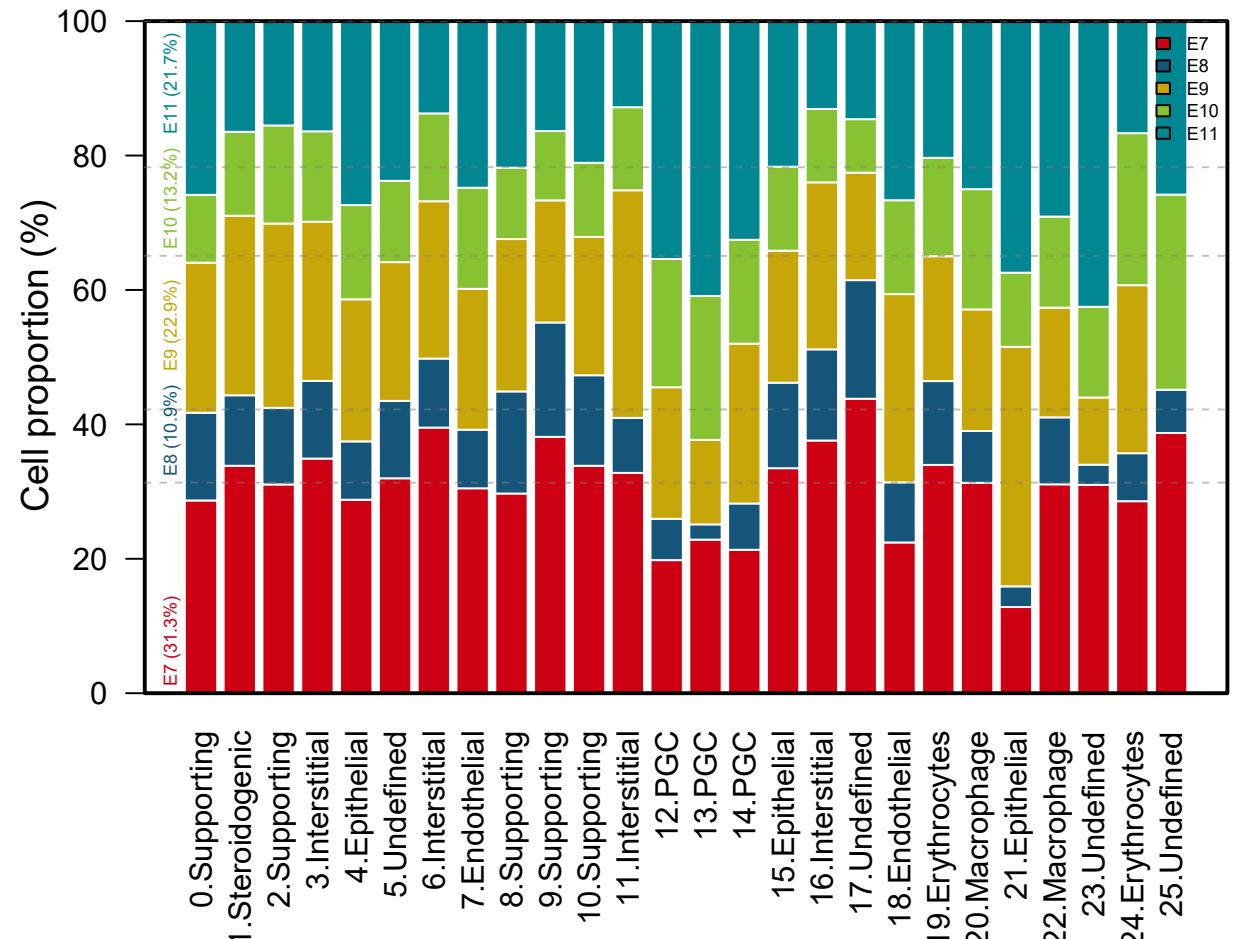

E

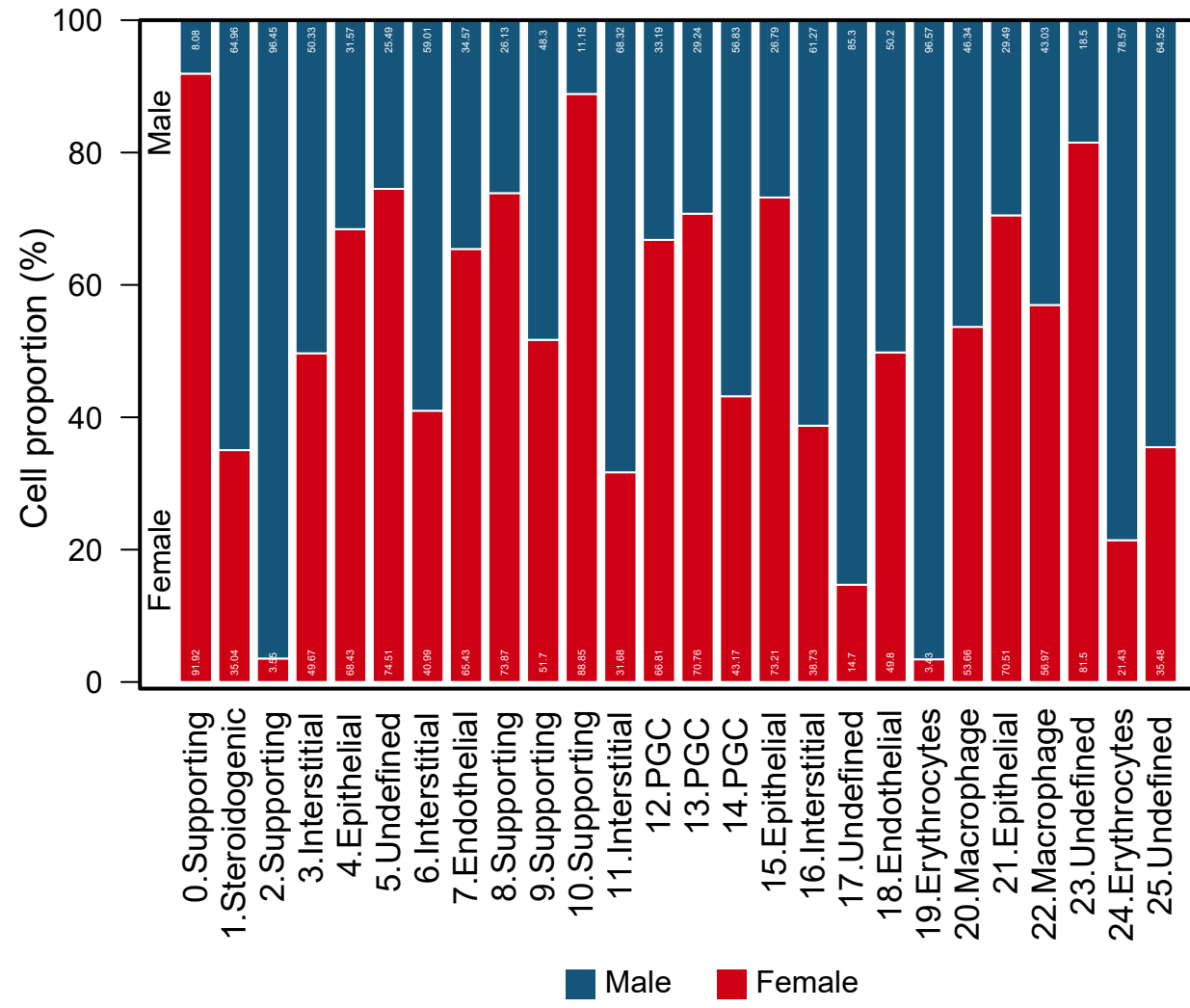

C

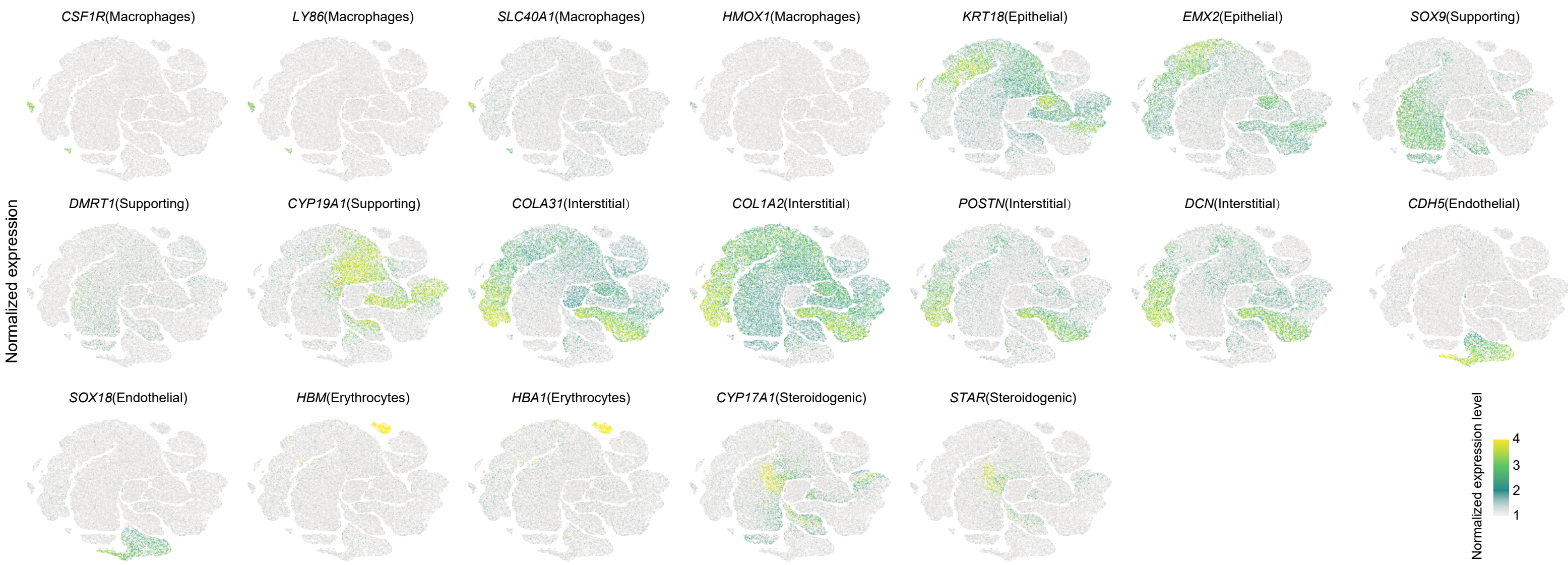

F

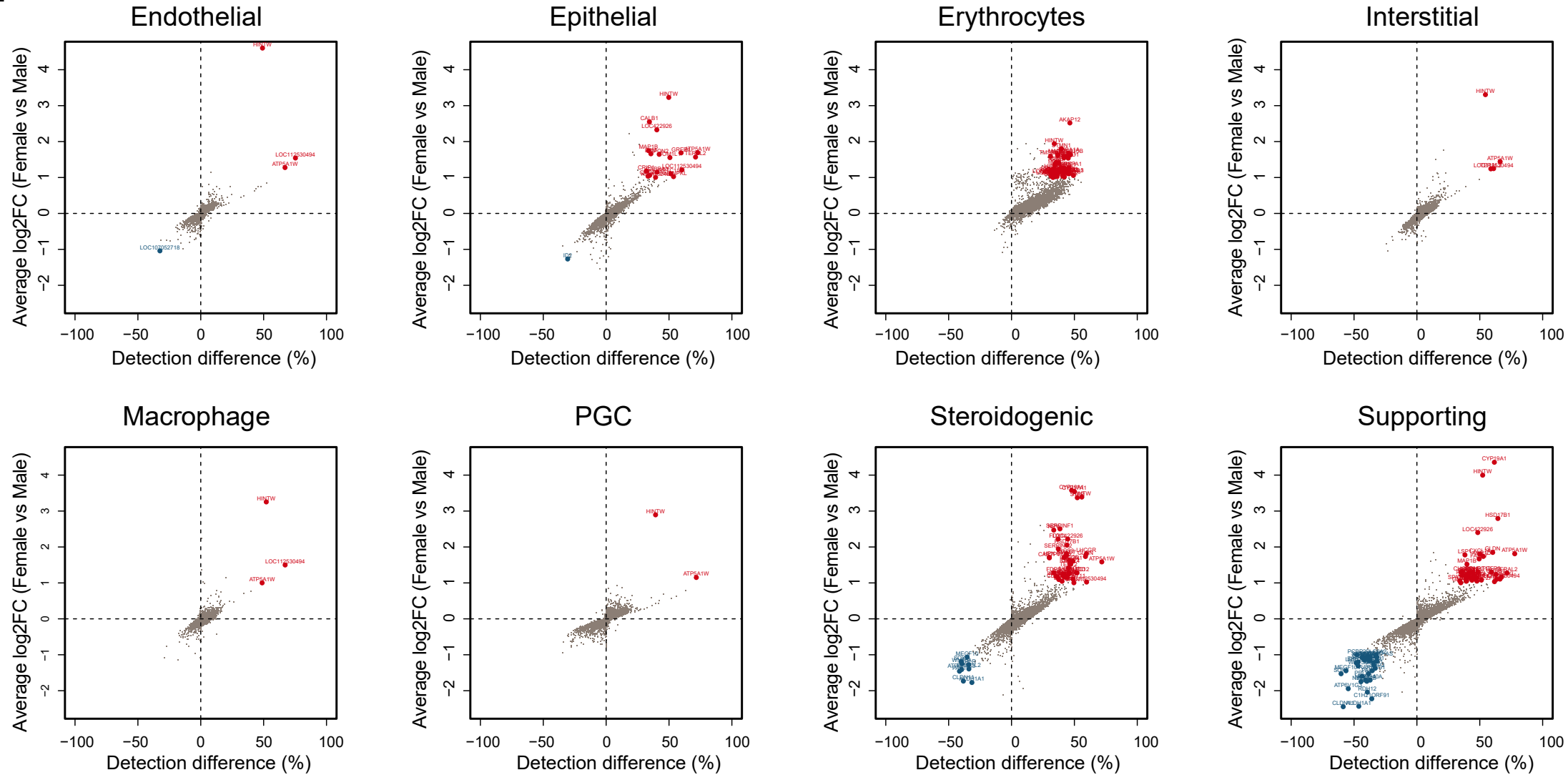

Figure S2

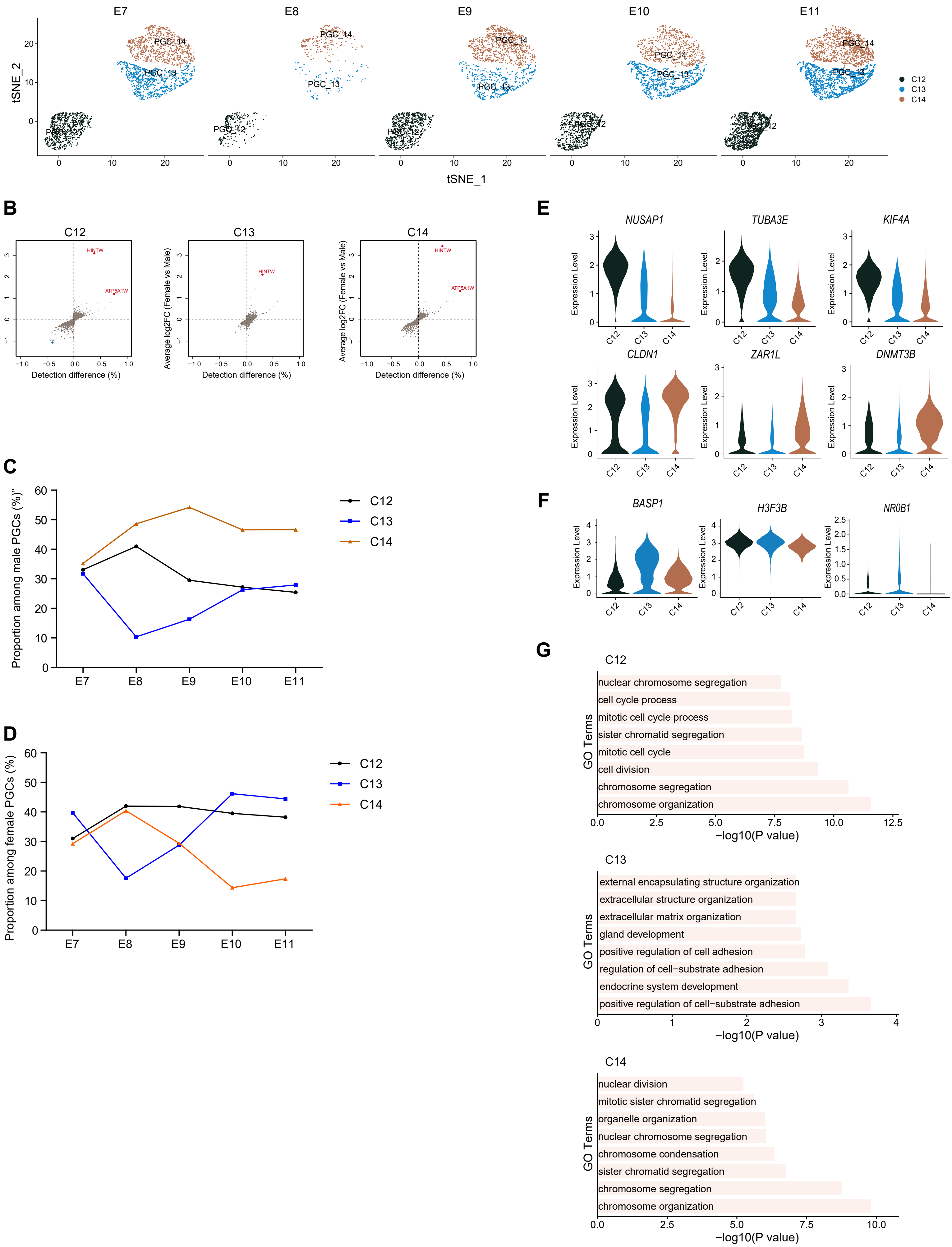
A
E7
E8
E9
E10
E11
tSNE_2
tSNE_1
PGC_14
PGC_13
PGC_12
C12
C13
C14
B
C12
C13
C14
Average log2FC (Female vs Male)
Detection difference (%)
HINTW
ATP5A1W
C
Proportion among male PGCs (%)"
D
Proportion among female PGCs (%)
E
NUSAP1
TUBA3E
KIF4A
CLDN1
ZAR1L
DNMT3B
Expression Level
F
BASP1
H3F3B
NR0B1
G
C12
nuclear chromosome segregation
cell cycle process
mitotic cell cycle process
sister chromatid segregation
mitotic cell cycle
cell division
chromosome segregation
chromosome organization
GO Terms
−log10(P value)
C13
external encapsulating structure organization
extracellular structure organization
extracellular matrix organization
gland development
positive regulation of cell adhesion
regulation of cell−substrate adhesion
endocrine system development
positive regulation of cell−substrate adhesion
C14
nuclear division
mitotic sister chromatid segregation
organelle organization
nuclear chromosome segregation
chromosome condensation
sister chromatid segregation
chromosome segregation
chromosome organization

Figure S3

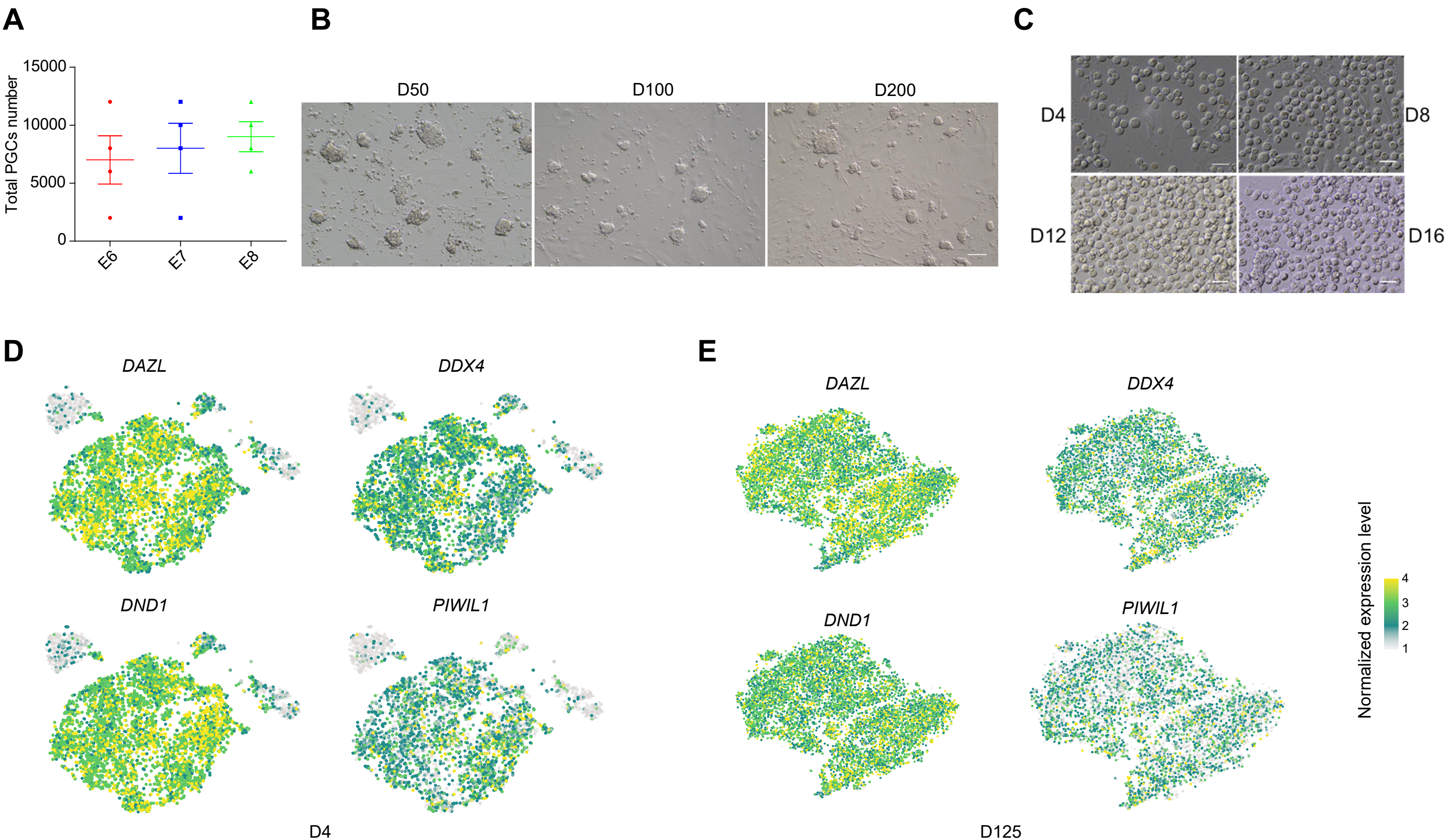

A
B
C
Total PGCs number
15000
10000
5000
0
E6
E7
E8
D50
D100
D200
D4
D8
D12
D16
D
E
DAZL
DDX4
DND1
PIWIL1
D4
DAZL
DDX4
DND1
PIWIL1
D125
Normalized expression level
4
3
2
1

Figure S4

A

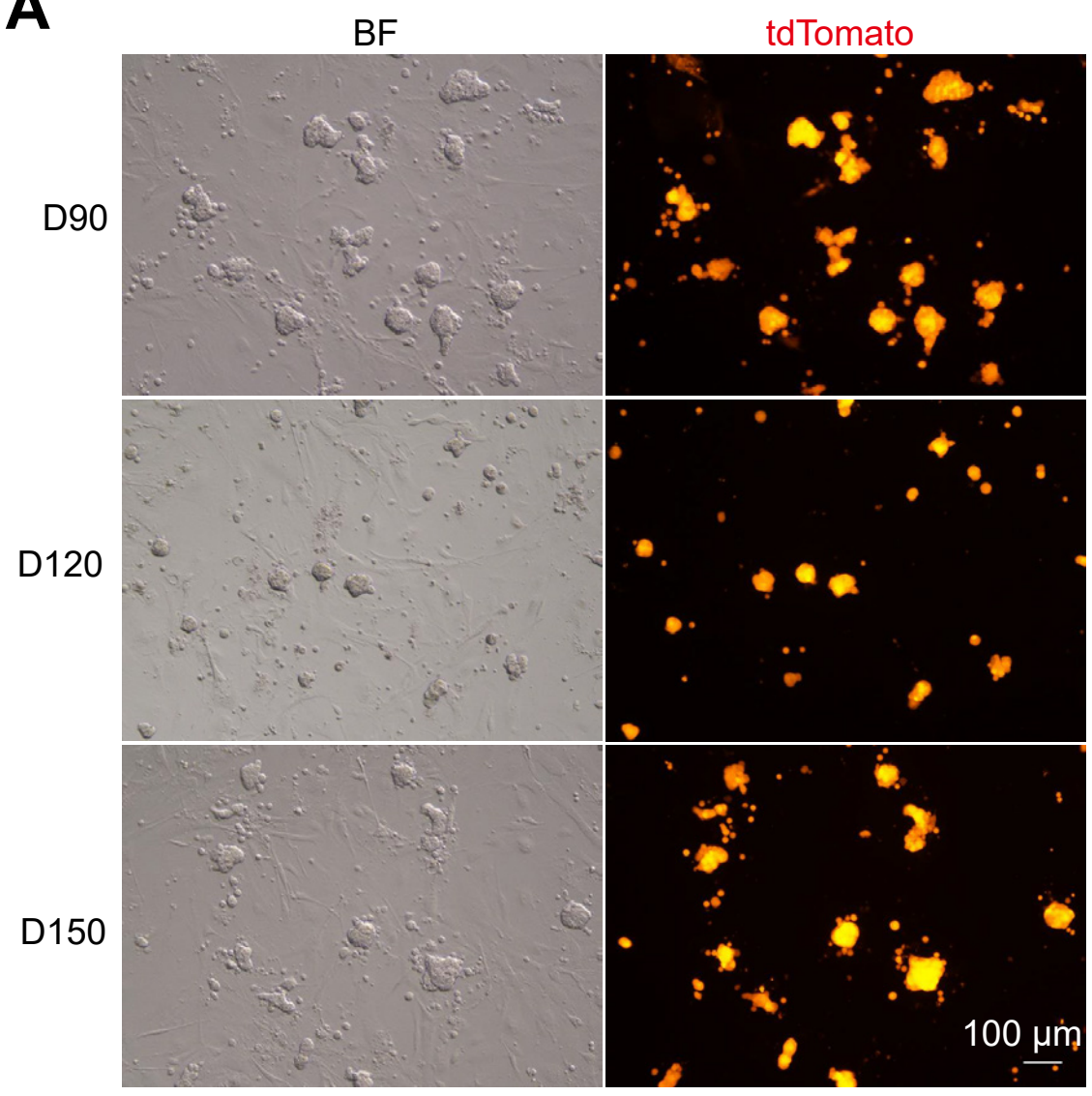

B

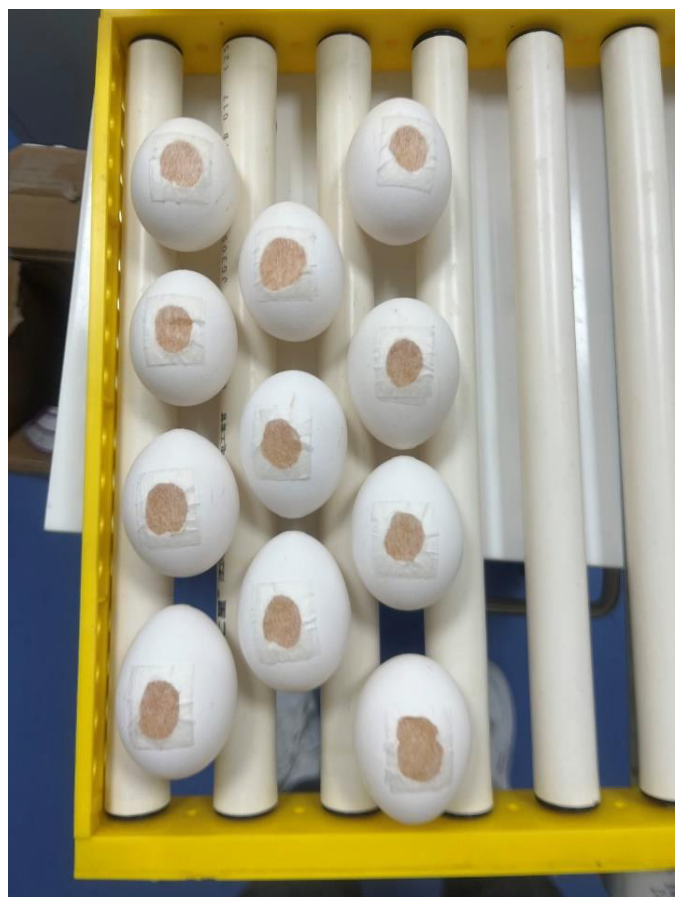

C

Cell type	No. of cells injected/embryo	No. of chimeric E7 embryos checked	Average chimerism ratio(%)
PGCs (D120-129)	2000-2500	11	0.12
PGCs (D130-139)	2000-2500	11	0.16
PGCs (D140-149)	2000-2500	5	0.14
PGCs (D150-159)	2000-2500	7	0.14

D

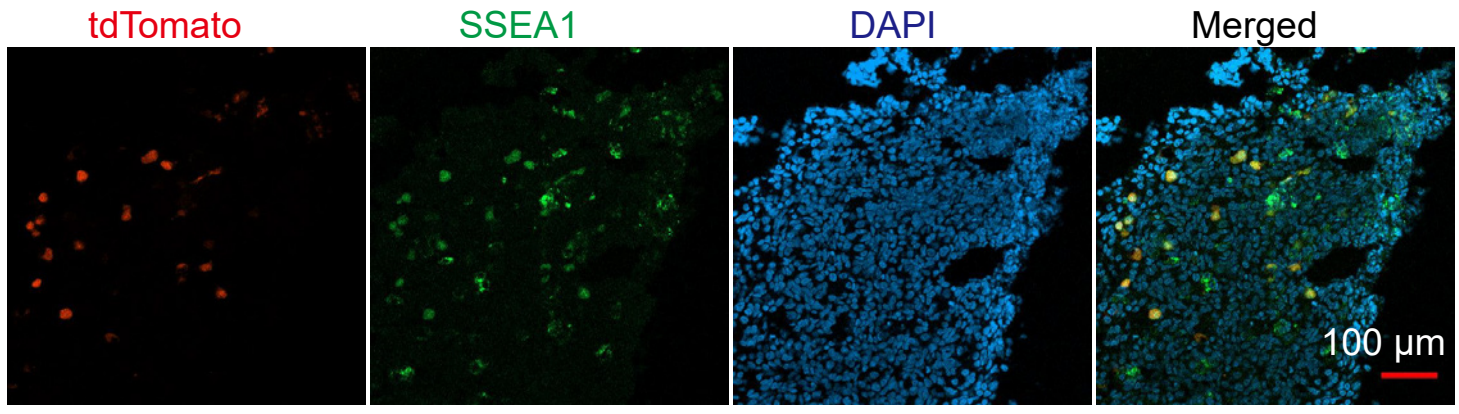

E

Cell type	No. of cells injected/embryo	No. of embryos incubated	Hatched chicken	Chimeric chicken
PGCs (D120)	2000-2500	15	3	1
PGCs (D132)	2000-2500	11	4	4
PGCs (D135)	2000-2500	9	1	1
PGCs (D136)	2000-2500	10	5	5

G

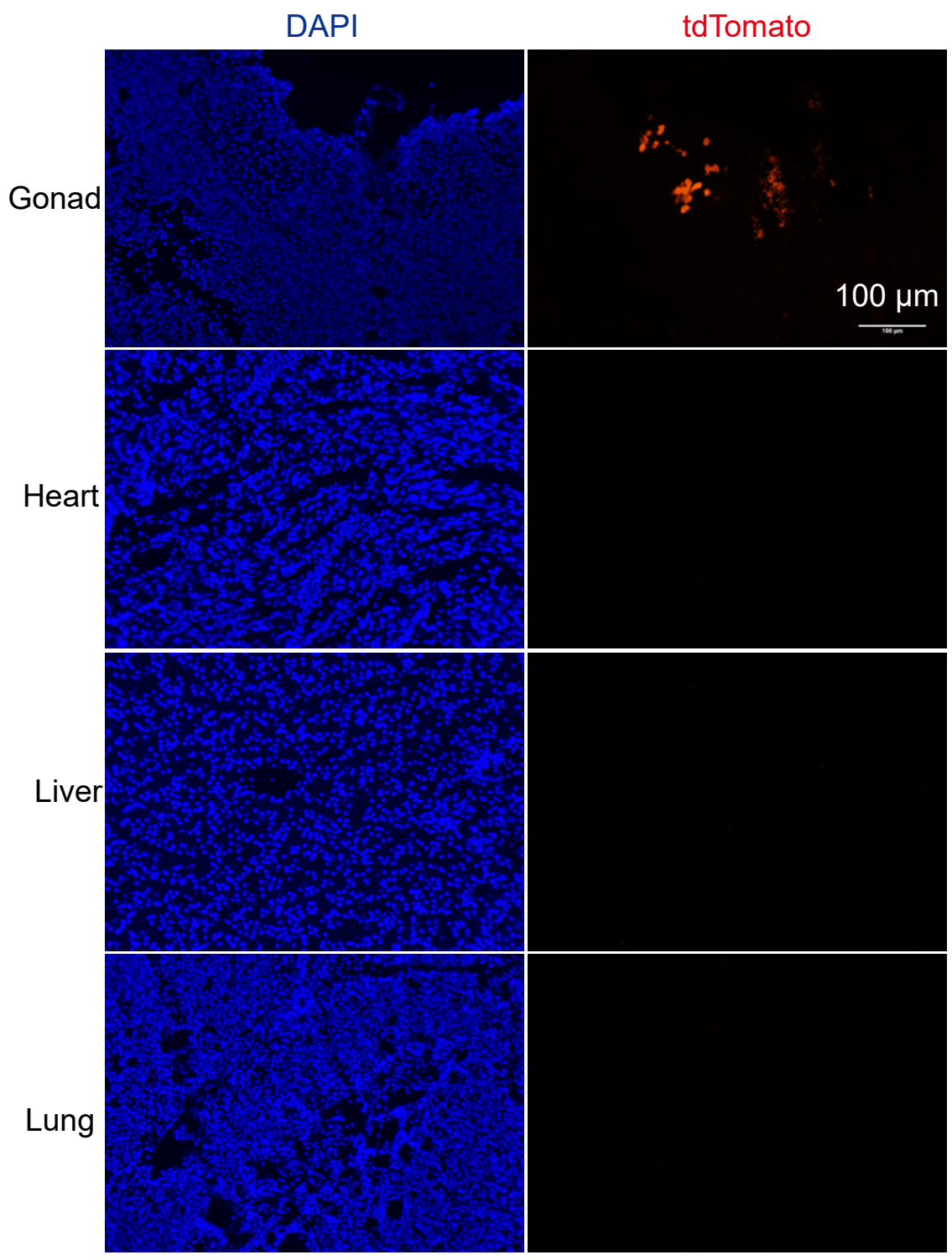

F

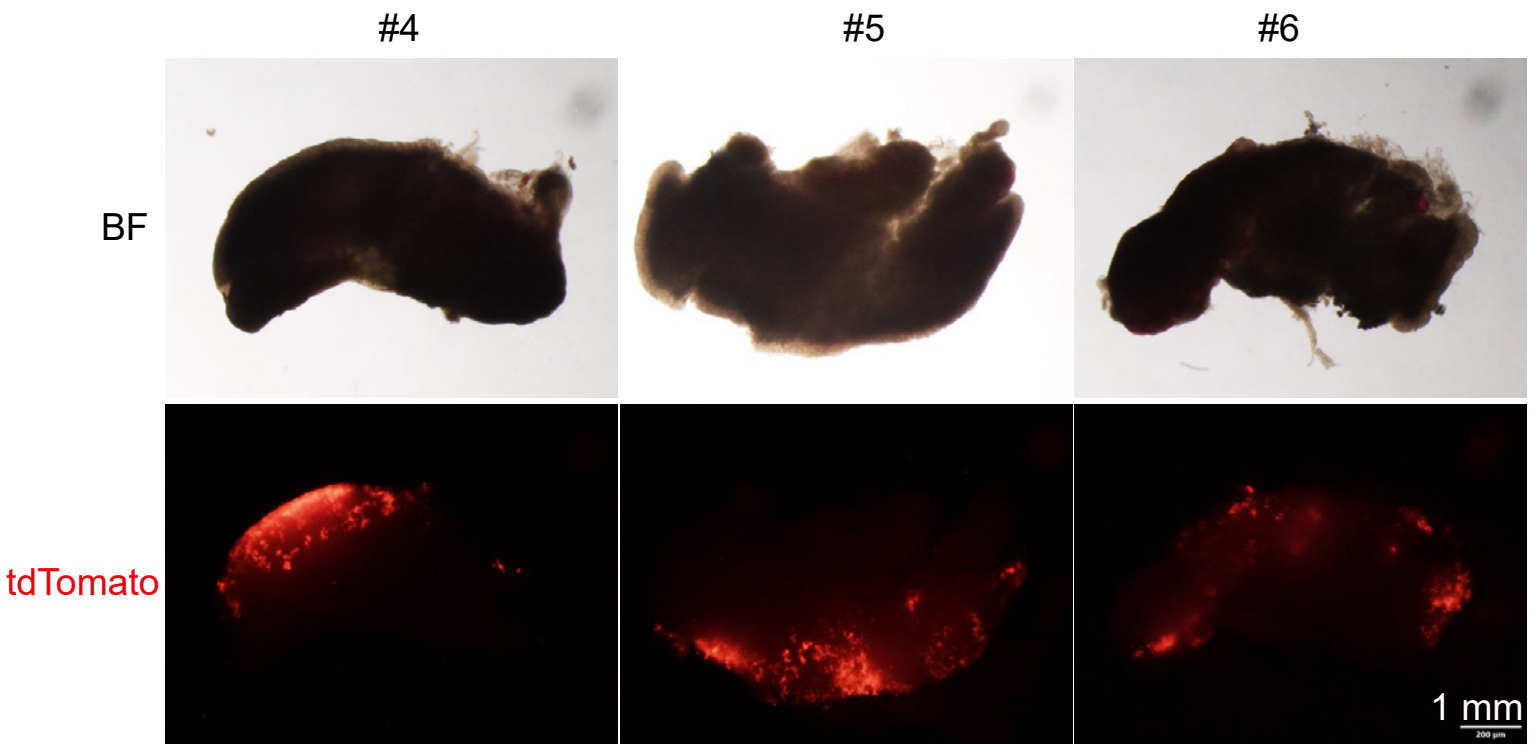

H

Stage	No. of cells injected/embryo	No. of chimeric embryos checked	Average chimerism ratio(%)
E9	2000-2500	6	0.27
P1-P3	2000-2500	6	0.69

I

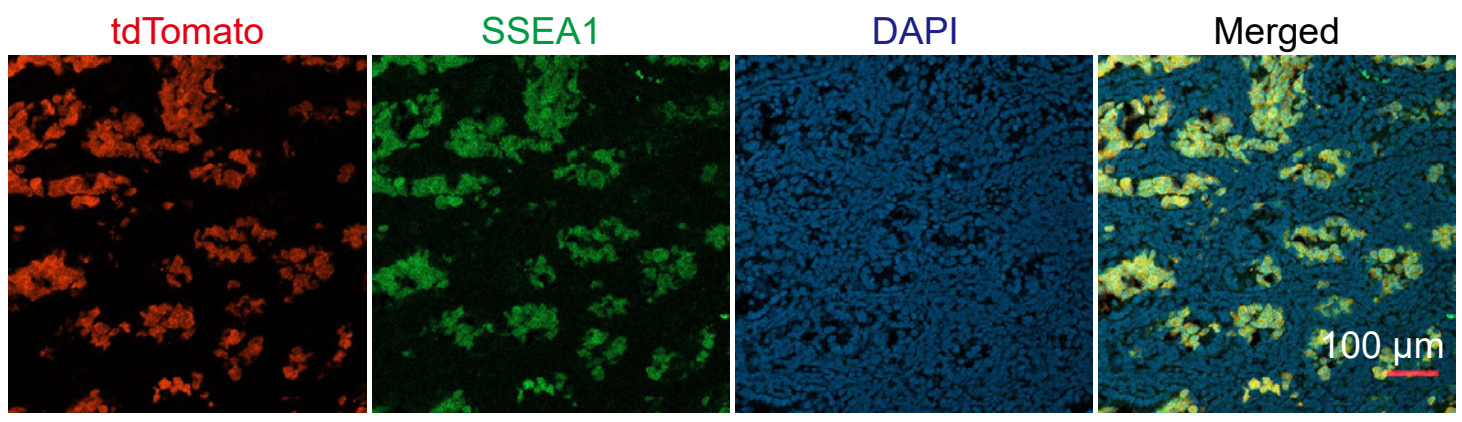

Figure S5

A

Cell line	Receive stage	No. of cells collected
#3	E7	60
#4	E7	100
#5	E7	700
#11	E7	500

B

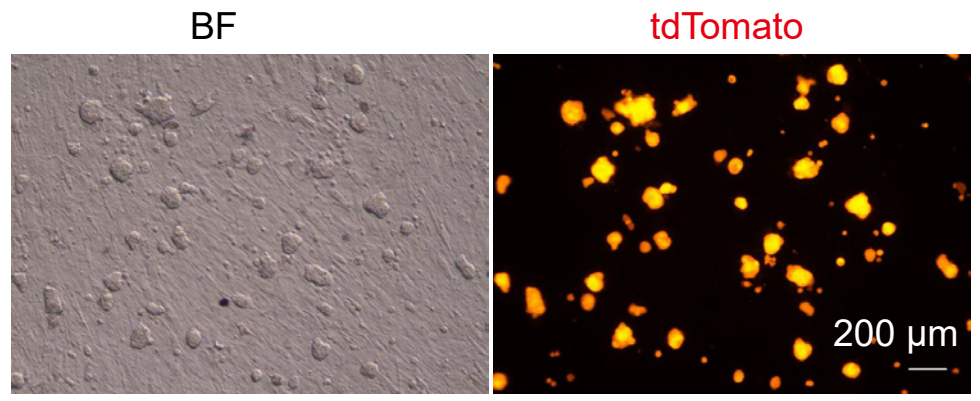

C

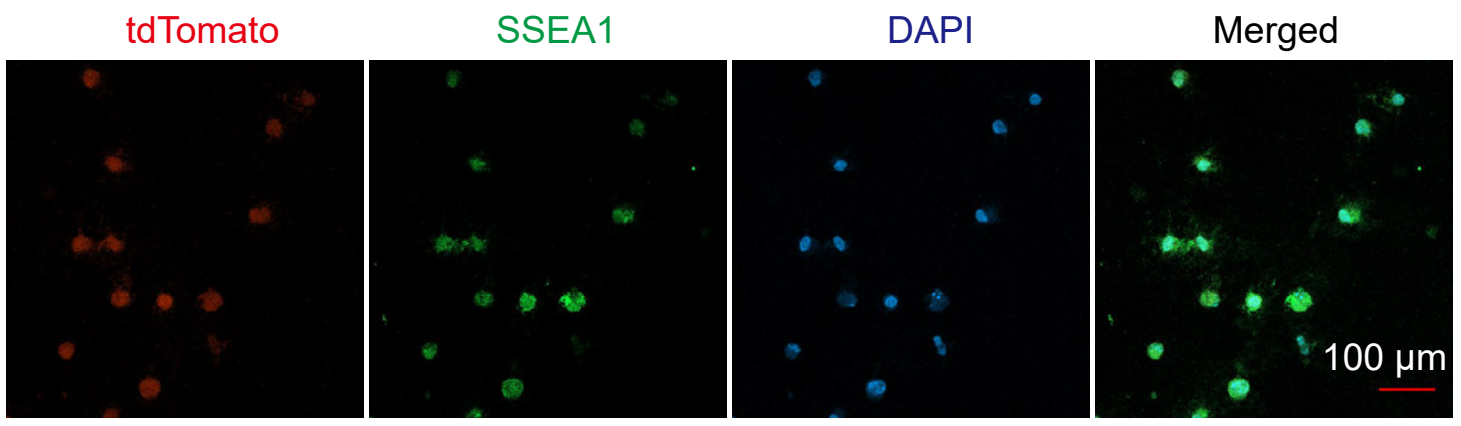

D

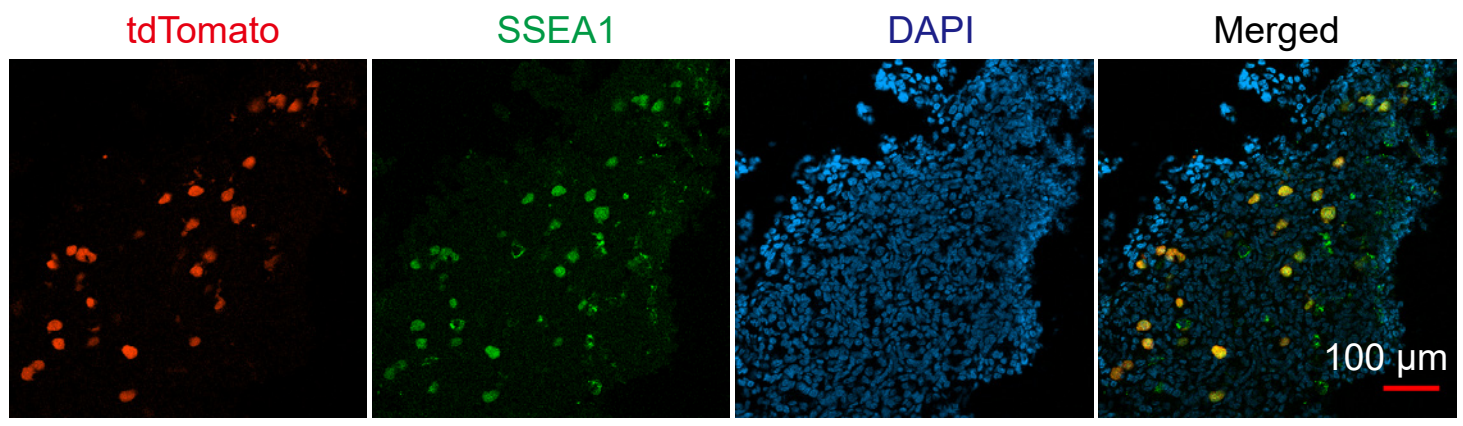

E

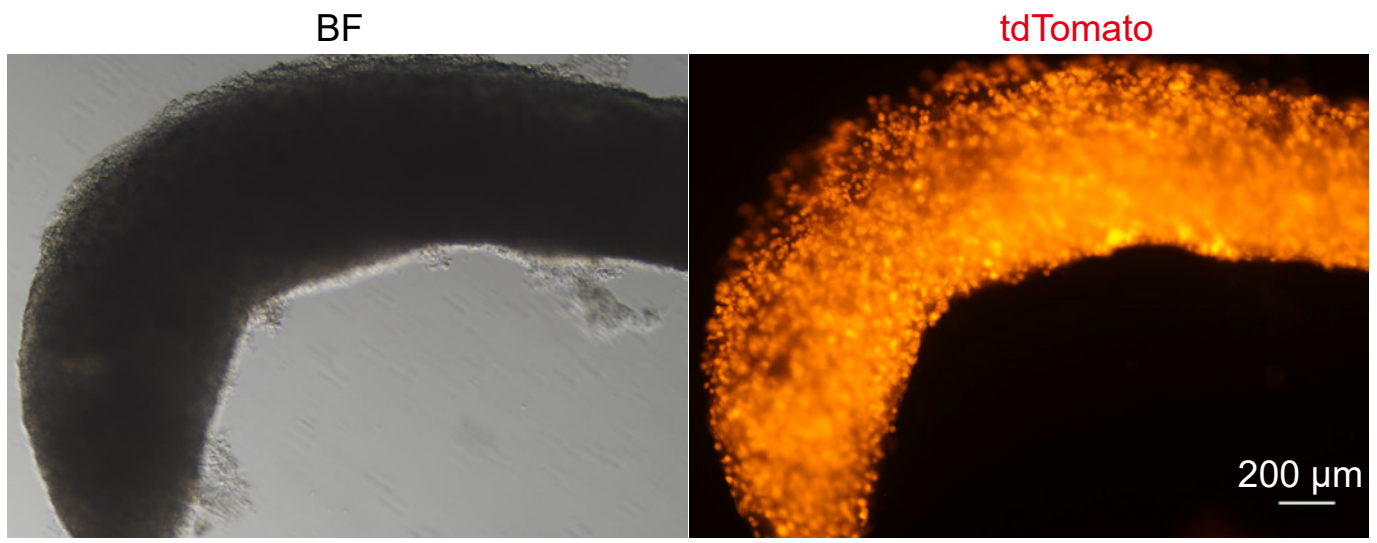

Supplement: Supplementary file 1 [file 18_2026_6331_MOESM2_ESM.pdf]
