# Supplementary material for: Deciphering the heterogeneity of chicken gonadal primordial germ cells via single-cell RNA sequencing
Source: Cell Mol Life Sci. 2026 Jul 22;83(1):387. doi: 10.1007/s00018-026-06331-w (PMC13680580; doi:10.1007/s00018-026-06331-w)
Supplement: Supplementary file 2 [file 18_2026_6331_MOESM1_ESM.docx]

**Supplementary Information**

**Supplementary figure legends**

**Supplementary Fig. S1. Single-cell RNA-seq analysis of chicken gonads.**

(A) Schematic workflow showing single-cell capture, cDNA library construction, and RNA sequencing of chicken gonadal cells collected from stages E7 to E11 embryos.

(B) Summary of scRNA-seq datasets generated from stages E7 to E11 gonadal cells and *in vitro*-cultured PGCs at D4 and D125.

(C) Feature plots showing the normalized expressions of marker genes used to annotate major gonadal somatic cell types, including macrophage, epithelial, supporting, interstitial, endothelial, erythrocyte, and steroidogenic cells.

(D) Stacked bar plot showing the proportions of stages E7 to E11 cells in each of the 26 clusters. Bar colors represent developmental stages from E7 to E11.

(E) Stacked bar plot showing the proportions of cells assigned to female or male origin in each of the 26 clusters. Red bars represent female cells and blue ones represents male cells.

(F) Differential expression plots comparing female and male cells within each major gonadal cell type. Red dots indicate genes upregulated in female cells, blue dots indicate genes upregulated in male cells, and gray dots indicate genes without marked sex-biased expression.

**Supplementary Fig. S2. Additional characterization of three transcriptionally distinct PGC subtypes in chicken gonads.**

(A) tSNE plots showing the distributions of the three PGC subtypes, C12, C13, and C14, across developmental stages from E7 to E11. PGCs are highlighted from the original whole-gonad tSNE embedding.

(B) Dot plots showing differentially expressed genes between female and male PGCs within each PGC subcluster. Red dots indicate upregulated genes in female cells (average log fold change > 0.58 and differential expression percentage > 10%), blue dots indicate genes upregulated in male cells (average log fold change < −0.58 and differential expression percentage < −10%), and gray dots indicate genes without marked sex-biased differential expression. Selected representative genes are labeled.

(C) Line plot showing the stage-wise proportions of C12, C13, and C14 among male PGCs from stages E7 to E11.

(D) Line plot showing the stage-wise proportions of C12, C13, and C14 among female PGCs from stages E7 to E11.

(E) Violin plots showing the normalized expressions of representative subtype-associated genes across C12, C13, and C14 PGCs. *NUSAP1*, *TUBA3E*, and *KIF4A* show higher expression in C12, whereas *CLDN1*, *ZAR1L*, and *DNMT3B* show higher expression in C14. Each violin shows normalized gene-expression distribution across cells in each PGC subcluster.

(F) Violin plots showing the normalized expressions of *BASP1*, *H3F3B*, and *NR0B1* across C12, C13, and C14 PGCs. These genes do not display clear subtype-restricted expression patterns in chicken PGCs.

(G) Bar plots showing representative Gene Ontology terms enriched among genes associated with C12, C13, and C14. The x-axis indicates −log10-transformed P values.

**Supplementary Fig. S3. Analysis of chicken gonadal PGCs cultured *in vitro*.**

(A) Numbers of PGCs isolated from stages E6, E7, and E8 gonads.

(B) Representative images of PGCs cultured *in vitro* for 50-200 days. Scale bar, 100μm.

(C) Representative images of PGCs cultured *in vitro* for 4, 8, 12, and 16 days. Scale bar, 50μm.

(D) Feature plots showing the normalized expressions of canonical PGC marker genes *DAZL*, *DDX4*, *DND1*, and *PIWIL1* in PGCs cultured *in vitro* for 4 days.

(E) Feature plots showing the normalized expressions of canonical PGC marker genes *DAZL*, *DDX4*, *DND1*, and *PIWIL1* in PGCs cultured *in vitro* for 125 days.

**Supplementary Fig. S4. Germline chimerism analysis following transplantation of *in vitro* cultured PGCs.**

(A) tdTomato-labeled PGCs cultured *in vitro* for 90-150 days. Scale bar, 100μm.

(B) Schematic representation of eggs handling and incubation after blood vessel injection.

(C) Summary of chimeric embryos at stage E7 after blood-vessel injection of PGCs cultured *in vitro* for 120-159 days.

(D) Immunofluorescence of recipient gonads at stage E7 showing tdTomato, SSEA1, and DAPI, and SSEA1 staining for PGC marker and DAPI staining for nuclei. Scale bar, 100 μm.

(E) Summary of chimeric chickens obtained after injection of *in vitro*-cultured PGCs.

(F) Representative gonads from female chimeric chickens imaged by bright field and fluorescence. Scale bar, 1 mm.

(G) Immunofluorescence analysis of gonad, heart, liver, and lung from chimeric chicken. Scale bar, 100μm.

(H) Summary of chimerism ratios in stage E9 gonads and chimeric chicken gonads after injection of PGCs cultured for 120-159 days.

(I) Immunofluorescence of P3 chimeric gonads showing tdTomato, SSEA1, and DAPI,and SSEA1 staining for PGC marker and DAPI staining for nuclei. Scale bar, 100 μm.

**Supplementary Fig. S5. Analysis of re-established gonadal PGC lines.**

(A) Summary re-established PGCs yield.

(B) Representative images of reestablished tdTomato-labeled PGCs cultured *in vitro*.

(C) Immunofluorescence staining of reestablished PGCs cultured *in vitro* showing tdTomato, SSEA1 and DAPI. Scale bar, 100 μm.

(D) Immunofluorescence of recipient gonads at stage E7 after secondary injection showing tdTomato, SSEA1, and DAPI. Scale bar, 100 μm.

(E) Representative stages E7 recipient gonads showing the tdTomato-positive cells after injection. Scale bar, 200 μm.

**Supplementary tables**

**Supplementary Table S1. Primer sequences used for RT-PCR**

| Primer name | Sequence (5′–3′) |
| --- | --- |
| NANOG-F | CAGCAGACCTCTCCTTGACC |
| NANOG-R | AAGCCCTCATCCTCCACAGC |
| POUV-F | GCCAAGGACCTCAAGCACAA |
| POUV-R | ATGTCACTGGGATGGGCAGA |
| CVH-F | GGGAAGATCAGTTTGGTGGA |
| CVH-R | GACAAAGAAAGGCTGCAAGG |
| DAZL-F | CGTCAACAACCTGCCAAGGA |
| DAZL-R | TTCTTTGCTCCCCAGGAACC |
| GAPDH-F | CACAGCCACACAGAAGACGG |
| GAPDH-R | CCATCAAGTCCACAACACGG |
